# Contribution to the knowledge of *Saprinus* Erichson, 1834 of forensic relevance from Lebanon (Coleoptera, Histeridae)

**DOI:** 10.3897/zookeys.738.21382

**Published:** 2018-02-19

**Authors:** Salman Shayya, Nicolas Dégallier, André Nel, Dany Azar, Tomáš Lackner

**Affiliations:** 1 Institut de Systématique, Évolution, Biodiversité, ISYEB - UMR 7205 – CNRS, MNHN, UPMC, EPHE, Muséum national d’Histoire naturelle, Sorbonne Universités, 57 rue Cuvier, CP 50, Entomologie F-75005, Paris, France; 2 Université Libanaise, Ecole Doctorale de Sciences et Technologies, Campus Universitaire de Rafic Hariri – Hadath, Lebanon; 3 Lebanese atomic energy commission, National Council of Scientific Research – Lebanon (CNRS-L); 4 State Key Laboratory of Palaeobiology and Stratigraphy, Nanjing Institute of Geology and Palaeontology, Chinese Academy of Sciences, Nanjing, Jiangsu 210008, China; 5 Lebanese University, Faculty of Sciences II, Department of Natural Sciences, P.O. Box: 26110217, Fanar – Matn, Lebanon; 6 120 rue de Charonne, 75011 Paris, France; 7 Bavarian State Collection of Zoology, Münchhausenstraße 21, 81247 Munich, Germany

**Keywords:** Carrion, key to species, Lebanon, peak activity, *Saprinus*

## Abstract

Many histerid beetles are necrophilous on carrion during both active and advanced stages of decomposition. In this study, 13 species of *Saprinus* were recorded on carrion from Lebanon, containing eight that are new for the Lebanese fauna. The following *Saprinus* species are newly recorded from Lebanon: 1) Saprinus (S.) caerulescens caerulescens (Hoffmann, 1803); 2) S. (S.) calatravensis Fuente, 1899; 3) S. (S.) chalcites (Illiger, 1807); 4) S. (S.) godet (Brullé, 1832); 5) S. (S.) maculatus (P. Rossi, 1792); 6) S. (S.) strigil Marseul, 1855; 7) S. (S.) submarginatus J. Sahlberg, 1913; and 8) S. (S.) tenuistrius sparsutus Solsky, 1876. The peak activity was recorded, key for the species is provided, and habitus images and male genitalia are illustrated in order to facilitate their taxonomic identifications. *Saprinus* species are diverse and common on animal carcass; they were likewise collected from a human cadaver in Lebanon. Preliminary comments on biology and distribution of the studied species are given. Our paper represents the first faunistic study on Histeridae from Lebanon. A rigorous research program regarding the biology of *Saprinus* in Lebanon and the neighbouring countries would greatly improve the knowledge of the diversity, activity, and possible forensic value of *Saprinus*.

## Introduction

Coleoptera (beetles) have proven to be useful in forensic investigations when fly larvae cannot be obtained or when they left the carcass and only beetles could be sampled (Zanetti et al. 2015). When examining the faunal succession of beetles on carcasses, the carrion beetles (Silphidae) are the first to be attracted, followed by the rove beetles (Staphylinidae) and clown beetles (Histeridae) (Su et al. 2013). Histerid beetles have received rather more attention in both ecological and taxonomic studies (three world catalogues published in the last 40 years, numerous revisions; for the references see Lackner et al. 2015). Regarding their presence on carrion, Histerids have been reported as the second after the Staphylinidae in the number of species on carrion during both the active and advanced stages of decomposition (Majka 2008; Su et al. 2013). Within the carrion community, histerid adults are regular visitors and they have been mentioned in forensic studies that use decomposing pig carcasses as experimental models as well as in real cases on human corpses (Bajerlein et al. 2011; Aballay et al. 2013). Histerids prey predominantly on dipteran eggs and larvae, though certain members of this family were also reported as predators on other beetles’ larvae and even to practice cannibalism at low preferred prey densities (Byrd and Castner 2000; Kovarik and Caterino 2016).

Histerids are unusual among beetles in having just two larval instars. In the first instar the head capsule and sclerites harden and the larva becomes capable of killing and feeding on soft-bodied insects. Mandibles and terminal palpal segments darken and become visible through the egg chorion just before eclosion (Kovarik and Caterino 2016). They undergo complete metamorphosis and their larvae are predacious and feed on insect immature stages. The average development of this family from egg to adult is 20 days at 30 °C (Fakoorziba et al. 2017). Our study focused on adults of *Saprinus* and immature stages were unfortunately not collected, albeit we admit that their presence might be informative for the determination of the post-mortem interval. Based on our data collected from pig carcasses, autopsies, and field trips, *Saprinus* species were the most common and diverse within the histerids. This paper aims to clarify the diversity, abundance, and association of *Saprinus* species with decomposing carcasses in Lebanon.

In the Palaearctic Region, 357 species of the Saprininae subfamily have been reported so far (Lackner et al. 2015). With 116 species, members of the genus *Saprinus* Erichson, 1834 are the most numerous. Regarding the countries neighbouring Lebanon, 47 species of the Saprininae are known from Syria, with 29 species recorded from Israel (Lackner et al. 2015). Among Saprininae, *Saprinus* species show similar relative diversities (Syria: 26 species; Israel: 18 species). Lebanon, with only 11 species of reported Saprininae, is the poorest of the three countries. Hitherto, only 5 species attributed to the genus *Saprinus*: S. (S.) aegialius Reitter, 1884; S. (S.) magnoguttatus Reichardt, 1926; S. (S.) prasinus prasinus Erichson, 1834; S. (S.) robustus Krása, 1944 (=S. (S.) vermiculatus Dahlgren, 1964) and S. (S.) subnitescens Bickhardt, 1909 have been confirmed from Lebanon (Lackner et al. 2015). In Lebanon, there is a dearth of faunistic studies on Coleoptera in general, and Histeridae are no exception. In our present study, we focused on the *Saprinus* species that were collected from cadavers, and provide a key to identification of *Saprinus* from Lebanon.

## Materials and methods

**Figure 1.**
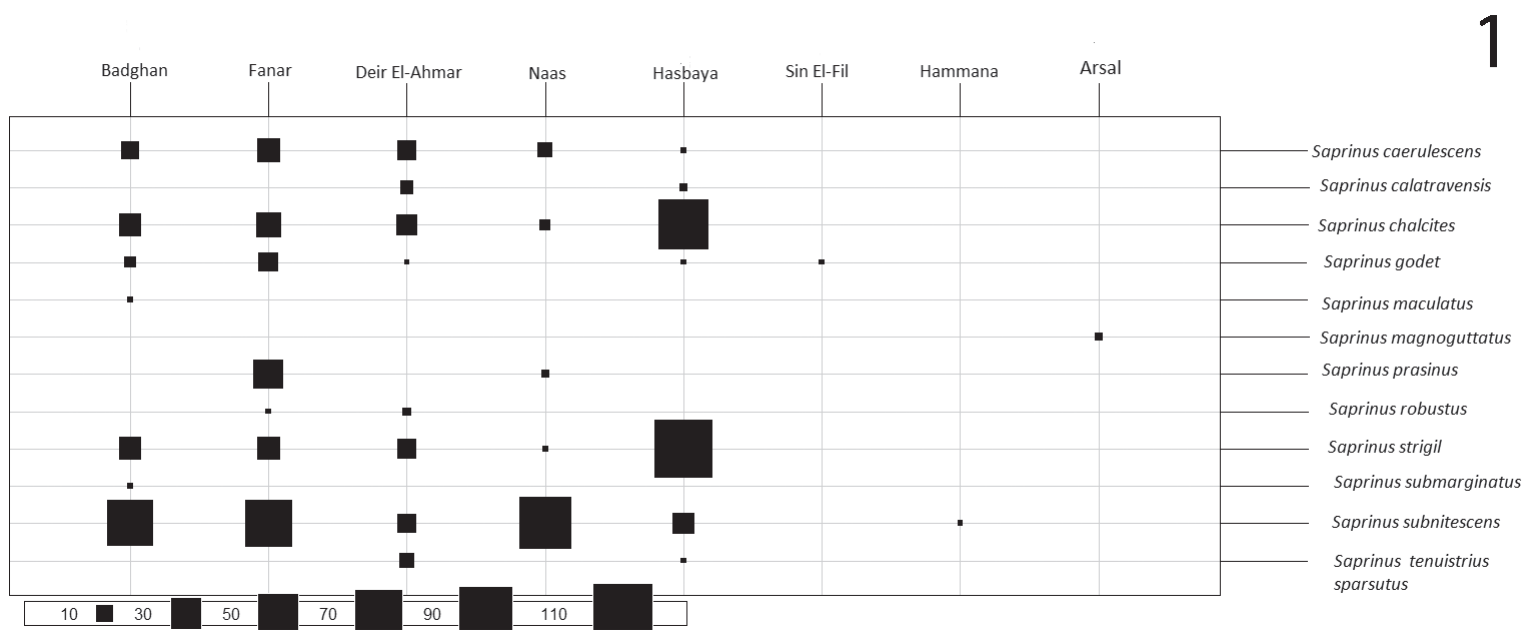
Abundance of *Saprinus* species in the localities of the present study.

The majority of specimens were collected on a daily basis during the decomposition process (Table 1). They were collected from the soil under five pig carcasses and 14 pitfall traps placed in their vicinity in addition to the sampling of two specimens during autopsy in Beirut, Beirut district, Beirut Governorate [33°53'N, 35°31'E] of a human body brought from Arsal, Baalbek-Hermel Governorate, Baalbek district [34°10'N, 36°25'E]. One pig carcass was placed exposed in each of the five different localities during different seasons (Fig. 1): 1) Badghan, Aley District, Mount Lebanon Governorate [33°46'4.24"N, 35°41'14.78"E] at an altitude of 1174 m during summer (June and July); 2) Fanar, Matn District, Mount Lebanon Governorate [33°52'44"N, 35°34'04"E] at an altitude of 250 m during spring (March and April); 3) Deir El-Ahmar, Baalbek-Hermel Governorate, Baalbek District [34°7'30.96"N, 36°7'22.04"E], at an altitude of 1040 m during summer (July) 3) Naas, Bikfaya Matn District, Mount Lebanon Governorate [33°54'42.4"N, 35°40'32.7"E] at the altitude of 1090 m during spring (April and May); and 5) Hasbaya, Natabtieh Governorate, Hasbaya District [33°23'52.35"N, 35°41.6'6.59"E], at an altitude of 750 m (July and August). In addition, a single pig head was placed in Fanar during the autumn season (September and October) and specimens were collected from pitfall traps surrounding it on a daily basis to compare the diversity of *Saprinus* during different weather conditions. Specimens from Hammana, Baabda District, Mount Lebanon Governorate [33°50'N, 33°44'E] at an altitude 1200 m and Sin El-Fil, Matn District, Mount Lebanon Governorate [33°52'N 35°32'E] at an altitude of 50 m were manually collected during field trips. Insects’ activity on the pig carcasses was also reported during the night.

**Table 1.** The sampled localities and the duration of the decomposition stages.

			**Stages of decomposition**			
**Locality**	**Coordinates**	**Season**	**Fresh**	**Bloat**	**Decay**	**Advanced decay**
Badghan	33°46'4.24"N, 35°41'14.78"E	Early summer	Day 1	Day 2–4	Day 5–9	Day 10–30
Fanar	33°52'44"N, 35°34'04"E	Spring	Day 1–4	Day 5–6	Day 7–16	Day 17–30
Deir El-Ahmar	34°7'30.96"N, 36°7'22.04"E	Summer	Day 1	Day 2–3	Day 4–5	Day 6–18
Naas	33°54'42.4"N, 35°40'32.7"E	Spring	Day 1–2	Day 3–6	Day 7–14	Day 15–30
Hasbaya	33°23'52.35"N, 35°41.6'6.59"E	Summer	Day 1	Day 2–3	Day 4–6	Day 7–30
Hammana	33°50'N, 33°44'E	Summer	One day sampling			
Sin El-Fil	33°52'N, 35°32'E	Spring	One day sampling			
Arsal	34°10'N, 36°25'E	Autumn	Autopsy done in Beirut			

Four stages of decomposition were reported on the carcasses: 1) fresh stage; 2) bloat stage; 3) active decay stage; and, 4) advanced decay stage. Based on Matuszewski et al. (2010), the onset of the bloat stage was the first day when the inflation of the carcass was present. Before the inflation the carcass was considered fresh. The duration of the active decay was associated to the presence of Diptera larvae that fed on the carcasses. The onset of the advanced decay was assigned when Diptera larvae moved away to pupate, and soft tissues were absent on the carcasses (Table 1).

General observation and dissection were carried out using stereomicroscope Nikon SMZ1500. Without genital extraction, the males of *Saprinus* species can be usually recognized through the examination of the anterior tarsal setae, which are expanded and lamellate, whereas they are unexpanded and pointed in female. Often the males possess a longitudinal depression on the metaventrite and occasionally also a single or two tiny tubercles on the apical metaventral margin. Male genitalia were first macerated in 10 % KOH solution for about 3 hours, cleared in 80 % ethanol and macerated in lactic acid with fuchsine, incubated at 60 °C for another 30 minutes, and subsequently cleared in 80 % ethanol and then observed in α-terpineol in a small dish. Digital photographs of male genitalia were taken by a Nikon 4500 Coolpix camera and edited in Adobe Photoshop CS5. Genitalia drawings based on the photographs or direct observations were produced with the aid of Hakuba klv-7000 light box. Habitus photographs were taken by F. Slamka (Bratislava, Slovakia). Specimens were measured with an ocular micrometre. Due to the lack of phylogenetic information regarding the genus *Saprinus*, the taxa in our present paper are arranged alphabetically. For the morphological terminology the reader is referred to Ôhara (1994) and especially Lackner (2010). The general distribution of *Saprinus* species is extrapolated from Mazur (2011). Whenever Mazur (2011) does not list a given species from Lebanon, while Lackner et al. (2015) report it, we specifically mention it. Specimens were identified using the key of Kryzhanovskij and Reichardt (1976) as well as comparing them with reliably identified voucher specimens deposited in the collection of T. Lackner. All synonymies of species listed here are according to Lackner et al. (2015), with the exception of Saprinus (S.) certus Lewis, 1888, which was transferred from the synonymy with S. (S.) chalcites to the synonymy with S. (S.) frontistrius Marseul, 1885 and Saprinus (S.) lindrothi Dahlgren, 1968 that was synonymized with Saprinus (S.) prasinus Erichson, 1834 (Lackner and Leschen 2017).

## Results

Our field experiments on pig cadavers in five localities across Lebanon (Fig. 1) resulted in four histerid genera: *Saprinus* Erichson, 1834 (85.52 %), *Margarinotus* Marseul, 1854 (14.08 %), *Atholus* C.G. Thompson, 1859 (0.27 %), and *Hypocacculus* Bickhardt, 1914 (0.13 %). We collected two species of *Margarinotus*: Margarinotus (Ptomister) brunneus (Fabricius, 1775) and Margarinotus (Grammostethus) ruficornis (Grimm, 1852); one species of *Atholus*: *Atholus duodecimstriatus duodecimstriatus* (Schrank, 1781); and one species of *Hypocacculus*: Hypocacculus (Hypocacculus) metallescens (Erichson, 1834). The most abundant and diverse was the genus *Saprinus* of which 13 species were reported. Out of these, eight species are herein reported as new for Lebanon: 1) Saprinus (S.) caerulescens caerulescens (Hoffmann, 1803); 2) S. (S.) calatravensis Fuente, 1899; 3) S. (S.) chalcites (Illiger, 1807); 4) S. (S.) godet (Brullé, 1832); 5) S. (S.) maculatus (P. Rossi, 1792); 6) S. (S.) strigil Marseul, 1855; 7) S. (S.) submarginatus J. Sahlberg, 1913; and 8) S. (S.) tenuistrius sparsutus Solsky, 1876. This paper analyses the diversity of necrophilous *Saprinus* spp. collected from Lebanon and establishes possible association with the decomposition stages, which could provide more clues for the determination of minimum post-mortem interval (PMI)_min_.

### Diagnosis of the genus *Saprinus* Erichson, 1834 from the Palaearctic region

The monophyletic (based on the literature references as well as on the on-going molecular studies by T.L.) subfamily Saprininae Blanchard, 1845 is characterised by the presence of distinctive sensory apparatus situated inside their antennal clubs (for more details on this, as well as on the general diagnosis of the subfamily see Lackner 2010) and by the opened antennal cavities, not covered by prosternal ‘alae’. Among the Saprininae, the genus *Saprinus* comprises usually ovoid to elongate-oval metallic histerid beetles (although they can also be entirely black, or possess reddish or yellowish maculae on their elytra) of moderate to rather large size (2.50–10.00) for the family. From most other genera, members of *Saprinus* differ usually by widely interrupted frontal stria in the combination of absent prosternal foveae. For the extensive diagnosis of this genus see Lackner (2010).

### *Saprinus* Erichson, 1834, of Lebanon known hitherto

#### 1. Saprinus (Saprinus) aegialius

Taxon classificationAnimaliaColeopteraHisteridae

Reitter, 1884

Fig. 2

= Saprinus incognitus Dahlgren, 1964
= Saprinus therondi Auzat, 1931

##### Type locality.

Greece.

##### Distribution.

Slovakia, Southern Europe, Mediterranean subregion, Armenia, Iran (Mazur 2011). Reported from Lebanon by Lackner et al. (2015).

##### Biology.

The biology of *S. aegialius* is not adequately known, but the species is often found on carcasses. This species is often confused with *Saprinus immundus* (Gyllenhal, 1808), and it was absent from our sampling (therefore not shown on a distributional map).

**Figure 2.**
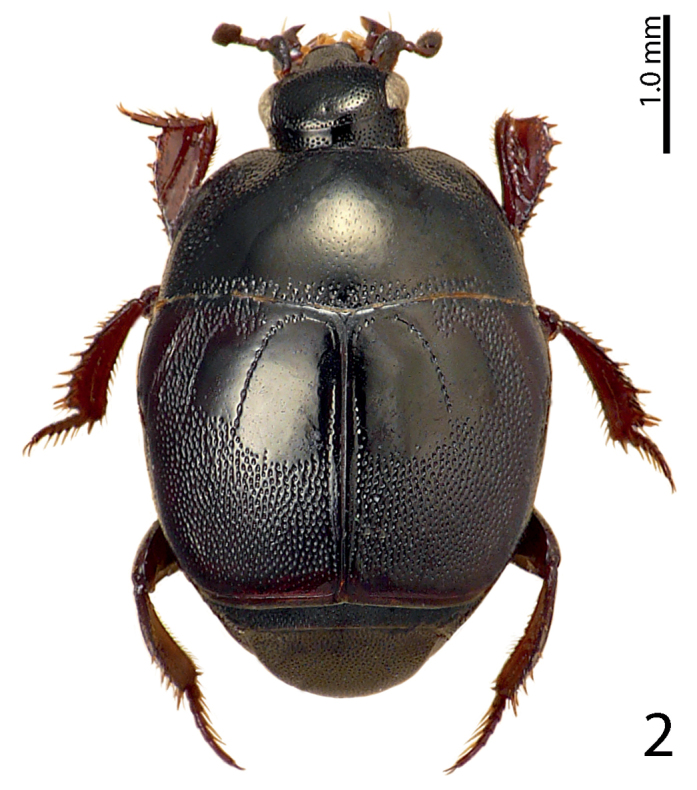
Saprinus (S.) aegialius Reitter, 1884 habitus, dorsal view.

#### 2. Saprinus (Saprinus) caerulescens caerulescens

Taxon classificationAnimaliaColeopteraHisteridae

(Hoffmann, 1803)

Figs 3 , 82 , 85a

Hister caerulescens Hoffmann, 1803
= Hister semipunctatus Fabricius, 1792
= Saprinus chobauti Auzat, 1926

##### Type locality.

Germany: Baden-Württemberg.

##### Distribution.

Southern Europe, Mediterranean subregion, Portugal (including the Azores Archipelago), Cape Verde Islands, Central Asia, introduced to Peru (Mazur 2011). Newly reported from Lebanon (Fanar, Deir El-Ahmar, Badghan, Naas, Hasbaya; Fig. 82).

##### Biology.

This species is found frequently on carrion, with a preference for larger carcasses (e.g., those of dogs, sheep, cattle, camels, etc. – T. Lackner pers. observ.). According to Kryzhanovskij and Reichardt (1976) S. (S.) caerulescens caerulescens was collected on rotten fish, where it preyed upon the beetles of the family Dermestidae. In our samples, we collected 47 specimens. S. (S.) caerulescens caerulescens was abundant during the active decay stage of carcass decomposition and coincides with the presence of Diptera larvae.

**Figure 3.**
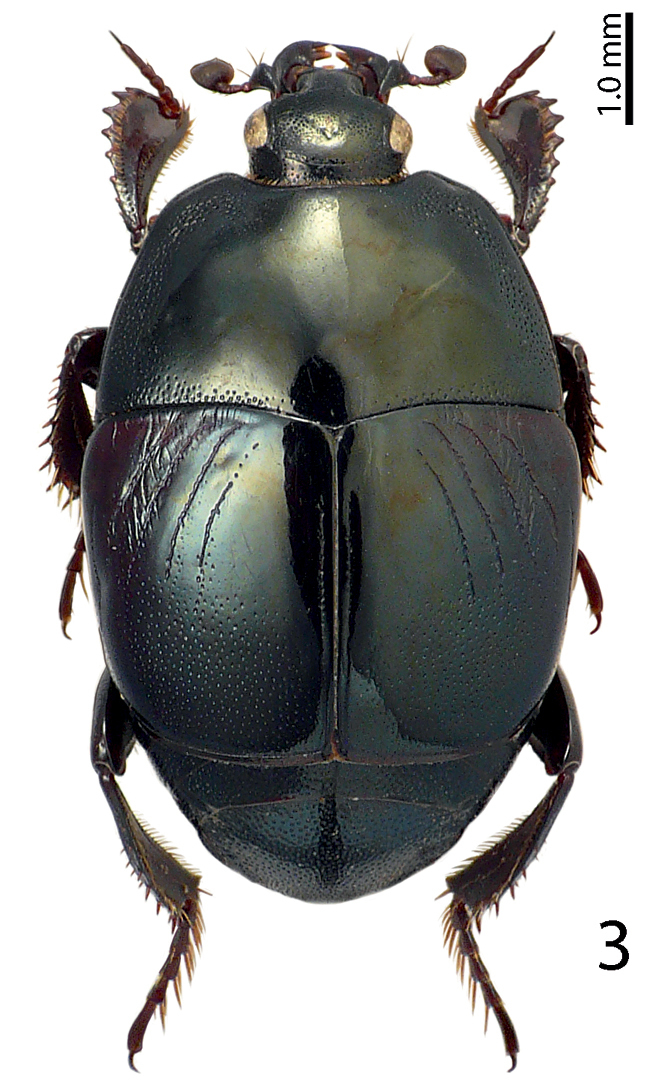
Saprinus (S.) caerulescens caerulescens (Hoffmann, 1803) habitus, dorsal view.

##### Comment.

This species has another subspecies S. (S.) caerulescens punctisternus Lewis, 1900 that occurs in China, Mongolia, Korean peninsula and Russian Far East (Mazur 2011).

#### 3. Saprinus (Saprinus) calatravensis

Taxon classificationAnimaliaColeopteraHisteridae

Fuente, 1899

Figs 4 , 15 , 16–24 , 82 , 85b

= Saprinus angoranus Bickhardt, 1911

##### Type locality.

Spain.

##### Distribution.

Mediterranean subregion, Arabian Peninsula, Central Asia (Mazur 2011). The distribution of this species is not sufficiently known due to its confusion with the morphologically similar species S. (S.) chalcites (Illiger, 1807) and, mainly *S. georgicus* Marseul, 1862. Newly reported from Lebanon (Deir El-Ahmar, Hasbaya; Fig. 82).

##### Biology.

*Saprinus calatravensis* is found on small and medium-sized carrion. According to Kryzhanovskij and Reichardt (1976) it prefers xerophilous landscapes and sandy soils. During our sampling eight specimens were collected of this species.

**Figure 4.**
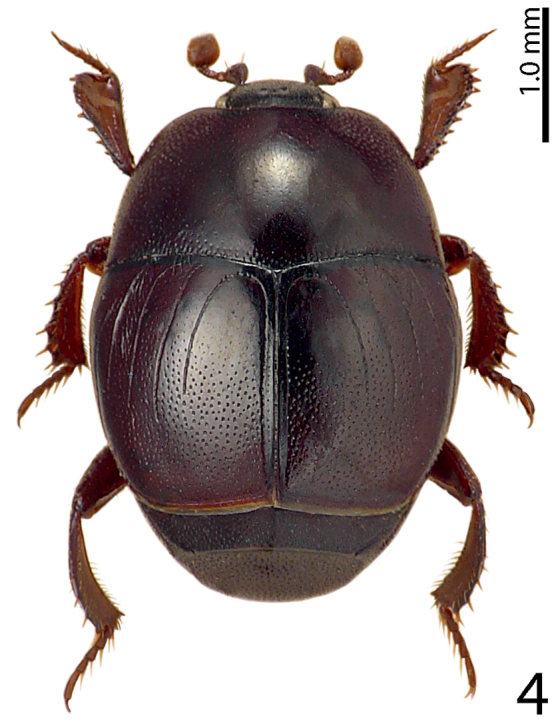
Saprinus (S.) calatravensis Fuente, 1899 habitus, dorsal view.

#### 4. Saprinus (Saprinus) chalcites

Taxon classificationAnimaliaColeopteraHisteridae

(Illiger, 1807)

Figs 5 , 25 , 26–34 , 82 , 86a

Hister chalcites Illiger, 1807
= Hister affinis Paykull, 1811
= Saprinus aerosus Normand & Thérond, 1952
= Saprinus melanocephalus Normand & Thérond, 1952
= Saprinus prolongatus Normand & Thérond, 1952
= Saprinus scapularis Normand & Thérond, 1952

##### Type locality.

Portugal.

##### Distribution.

Mediterranean subregion, Africa, Arabian Peninsula, Central Asia, India, Burma, Australia (Mazur 2011). Newly reported from Lebanon (Hasbaya, Fanar, Badghan, Deir El-Ahmar, Naas; Fig. 82).

##### Biology.

*Saprinus chalcites* is a widespread flying predator found on carcasses, rotting vegetable substances, as well as in dung. According to Kryzhanovskij and Reichardt (1976), large numbers of this species have also been found on flowering Stink lily (*Dracunculus vulgaris* Schott, 1832). In our samples, we collected 129 specimens, most abundantly during the active decay stage. Several specimens were likewise collected during the advanced decay stage after the departure of Diptera larvae from the carcasses.

**Figure 5.**
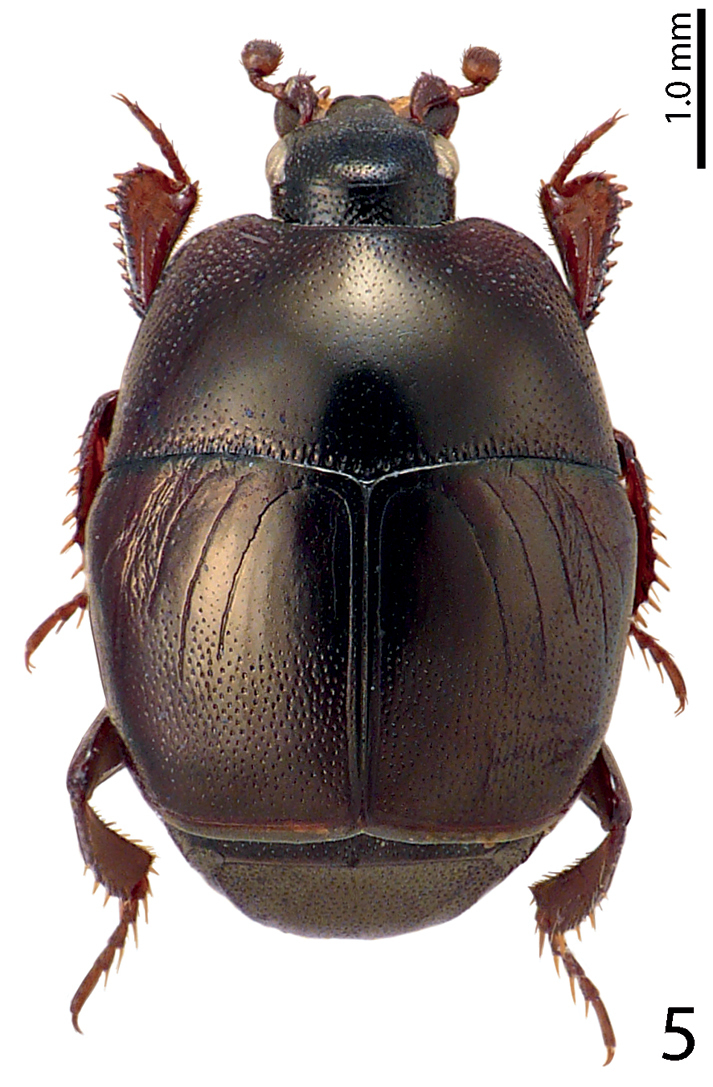
Saprinus (S.) chalcites (Illiger, 1807) habitus, dorsal view.

#### 5. Saprinus (Saprinus) godet

Taxon classificationAnimaliaColeopteraHisteridae

(Brullé, 1832)

Figs 6 , 35–43 , 82 , 86b

Hister godet Brullé, 1832
= Saprinus bitterensis Marseul, 1862
= Saprinus godetii Marseul, 1857 [emendation]
= Saprinus pseudolautus Reitter, 1904

##### Type locality.

Greece: Peloponnesus.

##### Distribution.

Spain, Portugal, southern France, Turkey, Georgia, Kazakhstan, Turkmenistan, Saudi Arabia (Mazur 2011). Reported from Azerbaijan, Greece, Italy, Mongolia, Uzbekistan and southern Russia by Tishechkin and Lackner (2017). Newly reported from Lebanon (Fanar, Badghan, Deir El-Ahmar, Hasbaya, Sin El-Fil; Fig. 82). In our samples 19 specimens were collected.

**Figure 6.**
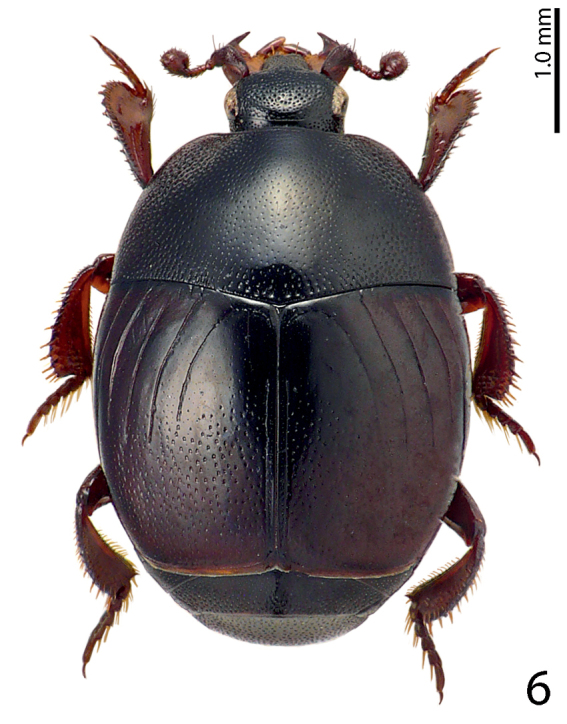
Saprinus (S.) godet (Brullé, 1832) habitus, dorsal view.

##### Biology.

Found on carrion (Kryzhanovskij and Reichardt 1976).

#### 6. Saprinus (Saprinus) maculatus

Taxon classificationAnimaliaColeopteraHisteridae

(P. Rossi, 1792)

Figs 7 , 83

Hister maculatus P. Rossi, 1792
= Saprinus obscuripennis J. Müller, 1899
= Hister personatus Fischer von Waldheim, 1823
= Saprinus pseudocruciata Auzat, 1920

##### Type locality.

Italy: Etrusca.

##### Distribution.

Slovakia, Hungary, south Europe, Turkey, Georgia, Azerbaijan, Cyprus, Iraq, Iran, Turkmenistan, Kazakhstan, Afghanistan (Mazur 2011). Newly reported from Lebanon (Badghan; Fig. 83).

##### Biology.

Found chiefly on cadavers, less commonly also in dung or human faeces (Kryzhanovskij and Reichardt 1976). In our samples a single specimen was collected.

**Figure 7.**
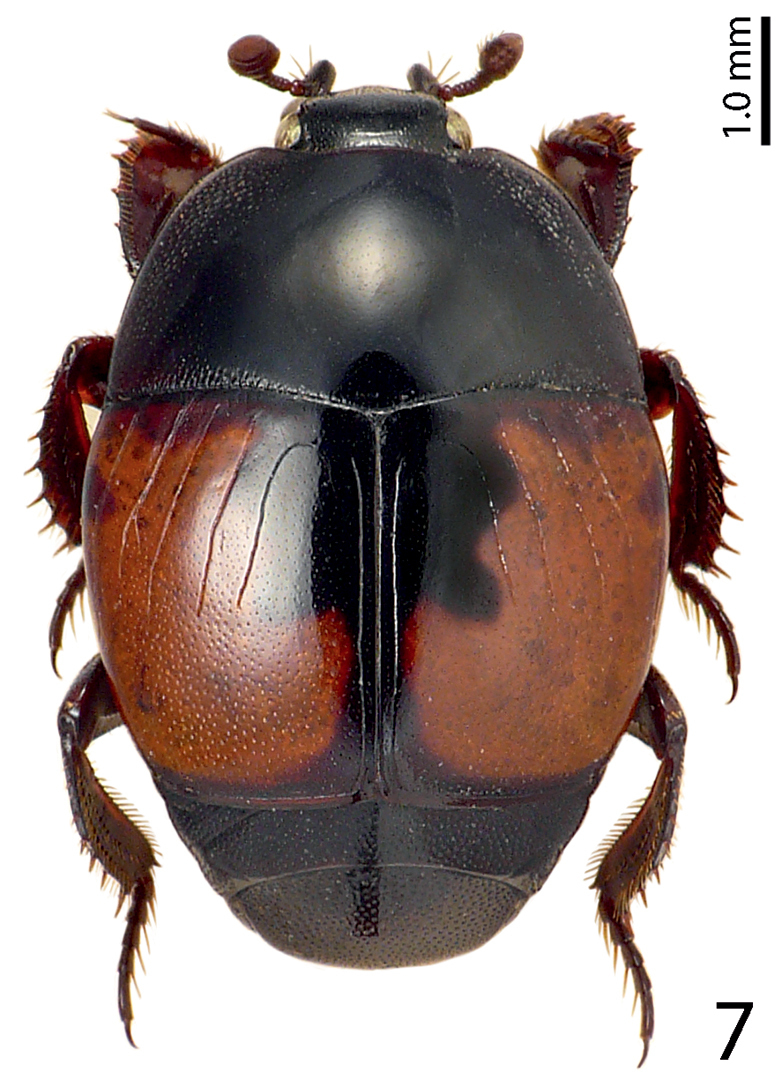
Saprinus (S.) maculatus (P. Rossi, 1792) habitus, dorsal view.

#### 7. Saprinus (Saprinus) magnoguttatus

Taxon classificationAnimaliaColeopteraHisteridae

Reichardt, 1926

Figs 8 , 83

##### Type locality.

Iran.

##### Distribution.

Turkey, Syria, Iraq, Azerbaijan, Lebanon (Mazur 2011).

##### Biology.

Reichardt (1941) reports this species from Azerbaijan as an inhabitant of mountain steppes (500 m); several specimens were also collected by pitfall trapping. In our samples, we collected two specimens from a human corpse that was in the active decay stage (Fig. 83).

**Figure 8.**
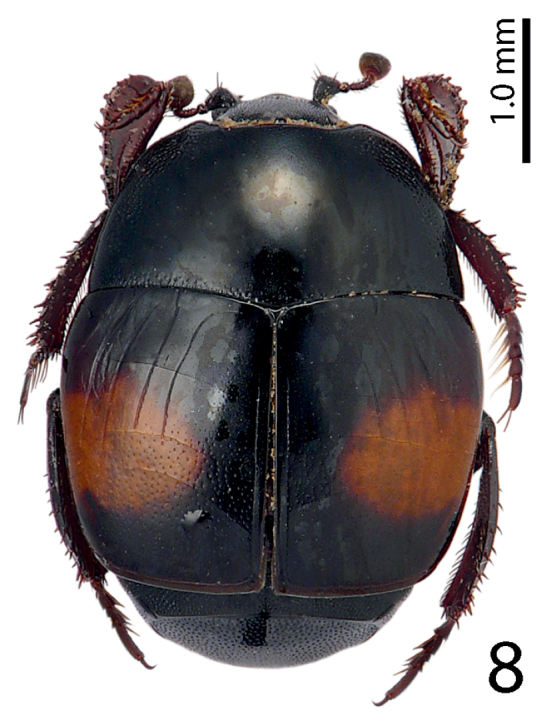
Saprinus (S.) magnoguttatus Reichardt, 1926 habitus, dorsal view.

##### Comment.

Lackner et al. (2015) wrongly attribute this species to G. Müller (1937).

#### 8. Saprinus (Saprinus) prasinus prasinus

Taxon classificationAnimaliaColeopteraHisteridae

Erichson, 1834

Figs 9 , 83 , 87a

= Saprinus lindrothi Dahlgren, 1968

##### Type locality.

Syria.

##### Distribution.

Italy: Sardinia, Greece: Crete, Bulgaria, Turkey, Armenia, Jordan (Mazur 2011). It is reported from Lebanon by Lackner et al. (2015). We collected this species from the following two localities, Fanar and Naas (Fig. 83).

##### Biology.

Widespread and frequent East-Mediterranean species found mostly on carrion (Rozner 2010). In our samples, 29 specimens were collected.

**Figure 9.**
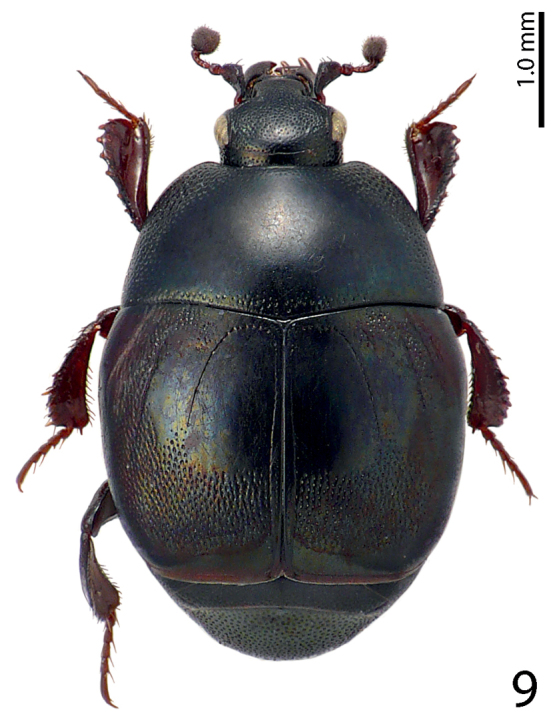
Saprinus (S.) prasinus prasinus Erichson, 1834 habitus, dorsal view.

##### Comment.

*Saprinus prasinus* has another subspecies S. (S.) prasinus aeneomicans G. Müller, 1960 that occurs in neighbouring Israel and Syria (Lackner et al. 2015). According to Kryzhanovskij and Reichardt (1976) this subspecies differs from the nominotypical one by a distinct bronze hue of the dorsum.

#### 9. Saprinus (Saprinus) robustus

Taxon classificationAnimaliaColeopteraHisteridae

Krása, 1944

Figs 10 , 44–52 , 83 , 87b

= Saprinus vermiculatus Dahlgren, 1964

##### Type locality.

Turkey: Ankara.

##### Distribution.

Greece: Crete, Turkey, Georgia, Syria, Lebanon (Mazur 2011). We report this species from the following two Lebanese localities: Deir El-Ahmar and Hasbaya (Fig. 83).

**Figure 10.**
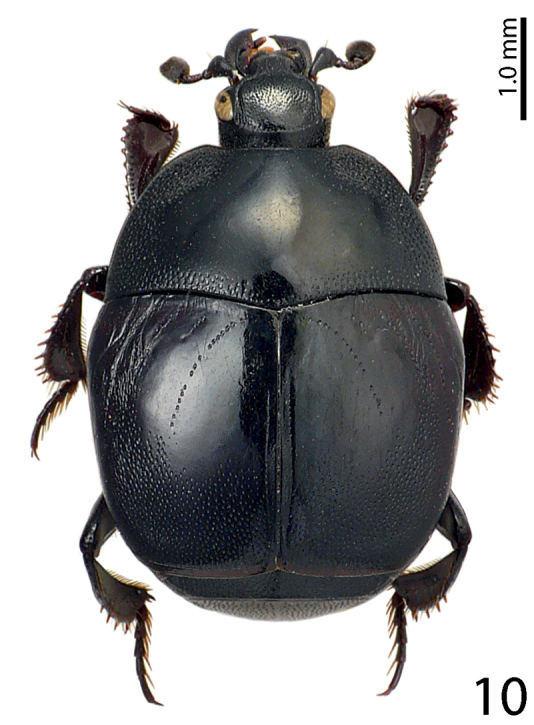
Saprinus (S.) robustus Krása, 1944 habitus, dorsal view.

##### Biology.

According to Anlaş et al. (2007) S. (S.) robustus is attracted to dung. In our samples, three specimens were collected.

#### 10. Saprinus (Saprinus) strigil

Taxon classificationAnimaliaColeopteraHisteridae

Marseul, 1855

Figs 11 , 84 , 88a

##### Type locality.

Ethiopia.

##### Distribution.

East and central Africa, Saudi Arabia, Turkey, Cyprus, Malta, Syria, Israel (Mazur 2011). Newly reported from Lebanon (Hasbaya, Badghan, Fanar, Deir El-Ahmar, Naas; Fig. 84).

**Figure 11.**
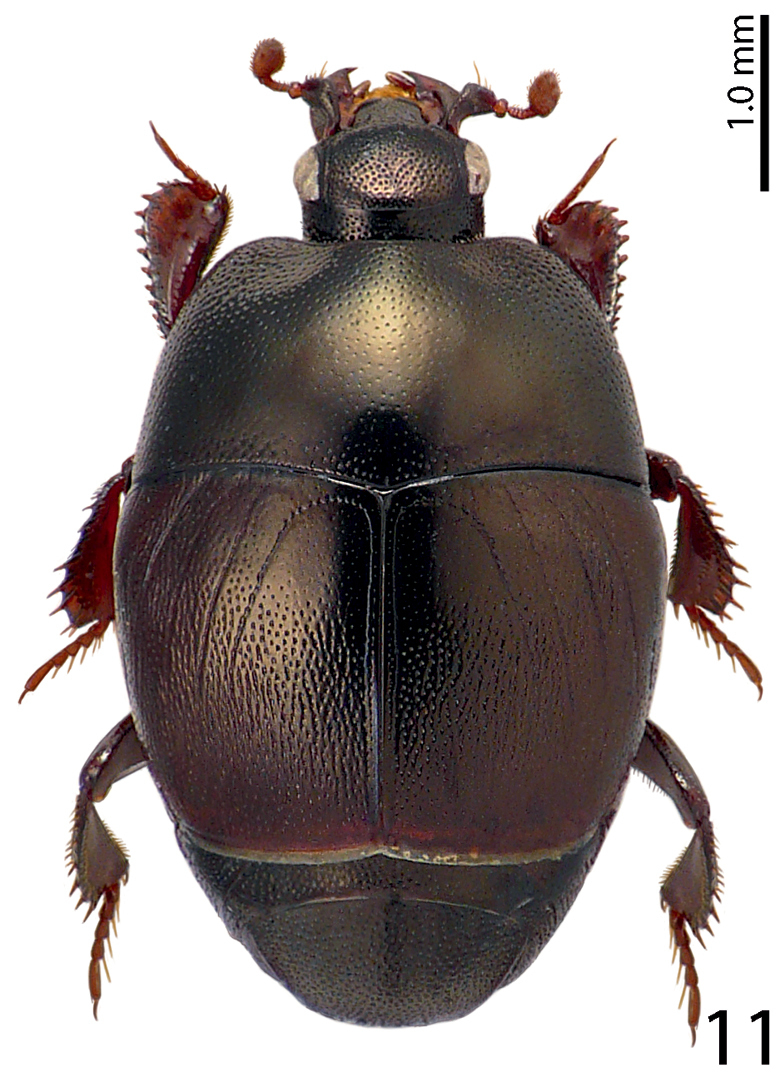
Saprinus (S.) strigil Marseul, 1855 habitus, dorsal view.

##### Biology.

Insufficiently known, according to our observations it is similar to its congeners. In our samples, we collected 149 specimens mainly during the active decay stage and several specimens in other stages of decomposition (Table 1).

#### 11. Saprinus (Saprinus) submarginatus

Taxon classificationAnimaliaColeopteraHisteridae

J. Sahlberg, 1913

Figs 12 , 53 , 54–62 , 84

##### Type locality.

Syria.

##### Distribution.

Algeria, Turkey, Israel, Armenia, Azerbaijan, Afghanistan, Iran (Mazur 2011). Newly reported from Lebanon (Badghan).

##### Biology.

According to Kryzhanovskij and Reichardt (1976), *S. submarginatus* is a typical inhabitant of xerophilous localities situated in higher elevations. During our sampling, a single specimen was collected. It was collected from altitude of 1174 m, during the summer season (June) from Badghan, Mount Lebanon where the rainfall average was 0 mm and the humidity 55.98 %, which would be in line with the above-mentioned observations of Kryzhanovskij and Reichardt (loc. cit.; Fig. 84).

**Figure 12.**
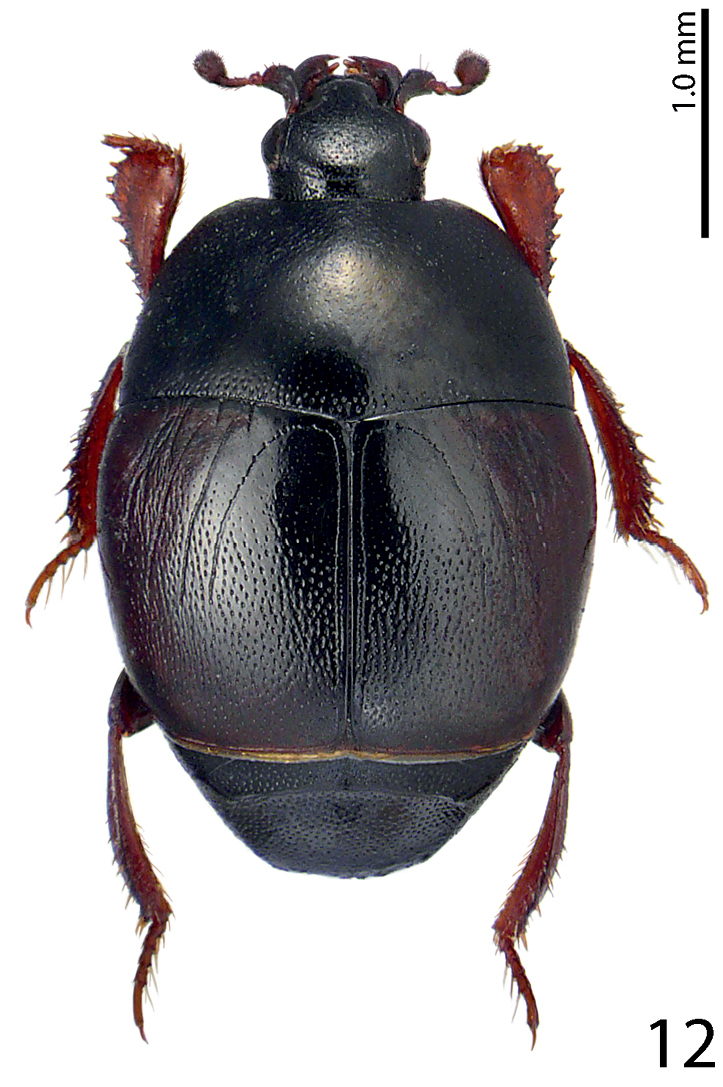
Saprinus (S.) submarginatus J. Sahlberg, 1913 habitus, dorsal view.

#### 12. Saprinus (Saprinus) subnitescens

Taxon classificationAnimaliaColeopteraHisteridae

Bickhardt, 1909

Figs 13 , 63 , 64–72 , 84 , 88b

= Saprinus fagniezi Auzat, 1912
= Saprinus lecontei Casey, 1916
= Saprinus meridionalis Ihssen, 1949
= Saprinus simulans J. Sahlberg, 1913

##### Type locality.

Hungary, Romania.

##### Distribution.

Central and south Europe, North Africa, Spain (including Canary Islands), Portugal (including Madeira), Turkey, central Asia, introduced to north America (Mazur 2011). From Lebanon already reported by Lackner et al. (2015). We herein report this species from the following Lebanese localities: Fanar, Badghan, Naas, Deir El-Ahmar and Hammana (Fig. 84).

**Figure 13.**
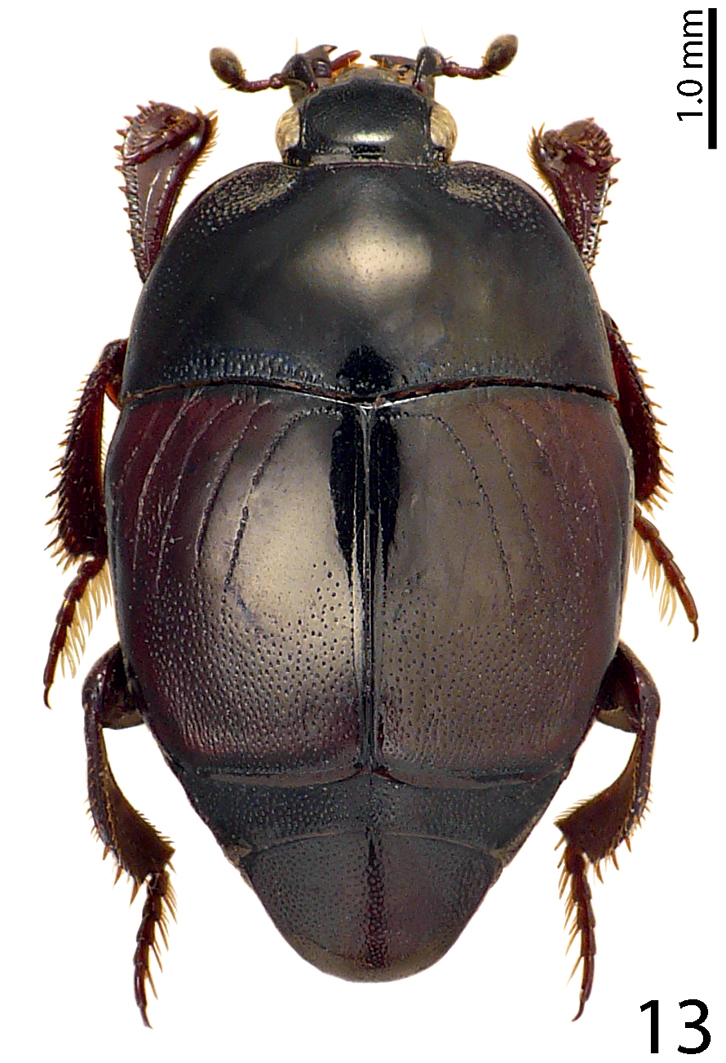
Saprinus (S.) subnitescens Bickhardt, 1909 habitus, dorsal view.

##### Biology.

A typical free-living volant predator found on carrion as well as in dung. In our samples we collected 242 specimens. Saprinus (S.) subnitescens was the most abundant species on the carcasses during both the active and advanced stage of decomposition.

#### 13. Saprinus (Saprinus) tenuistriussparsutus

Taxon classificationAnimaliaColeopteraHisteridae

Solsky, 1876

Figs 14 , 73–81 , 84 , 89

= Saprinus brunnensis A. Fleischer, 1883

##### Type locality.

Uzbekistan.

##### Distribution.

Central and South Europe, Central Asia, Mongolia, North China (Mazur 2011). Herein newly reported from Lebanon (Deir El-Ahmar, Hasbaya; Fig. 84).

##### Biology.

This species is found predominantly on carcasses. During our sampling eight specimens were collected.

**Figure 14.**
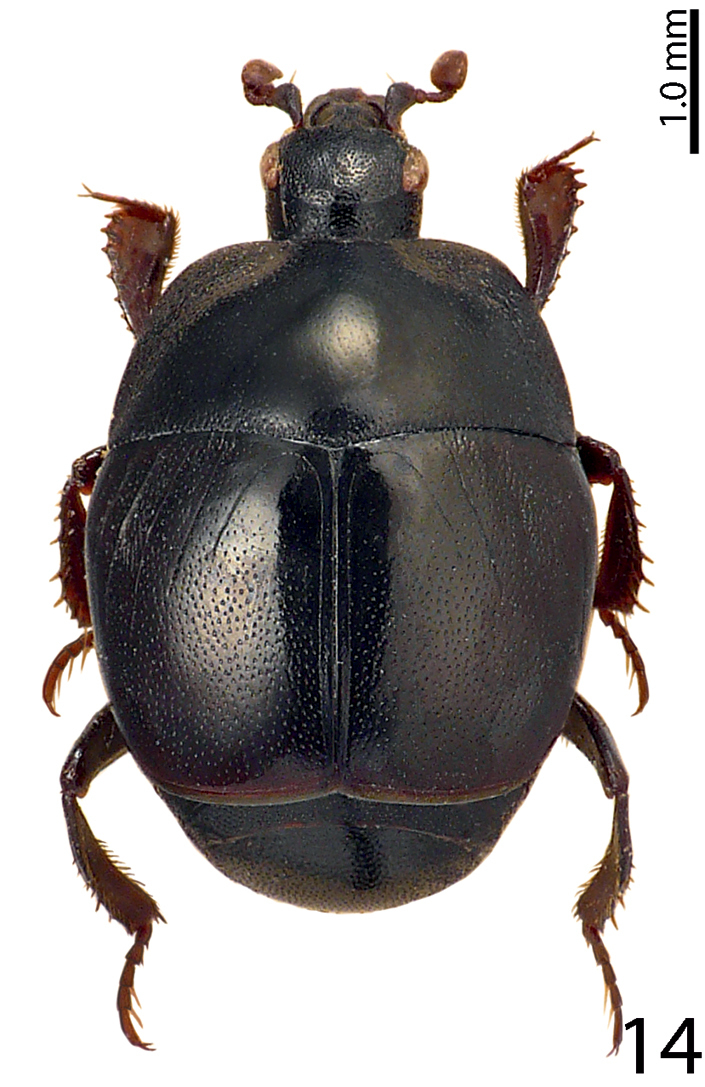
Saprinus (S.) tenuistrius sparsutus Solsky, 1876 habitus, dorsal view.

**Figure 15.**
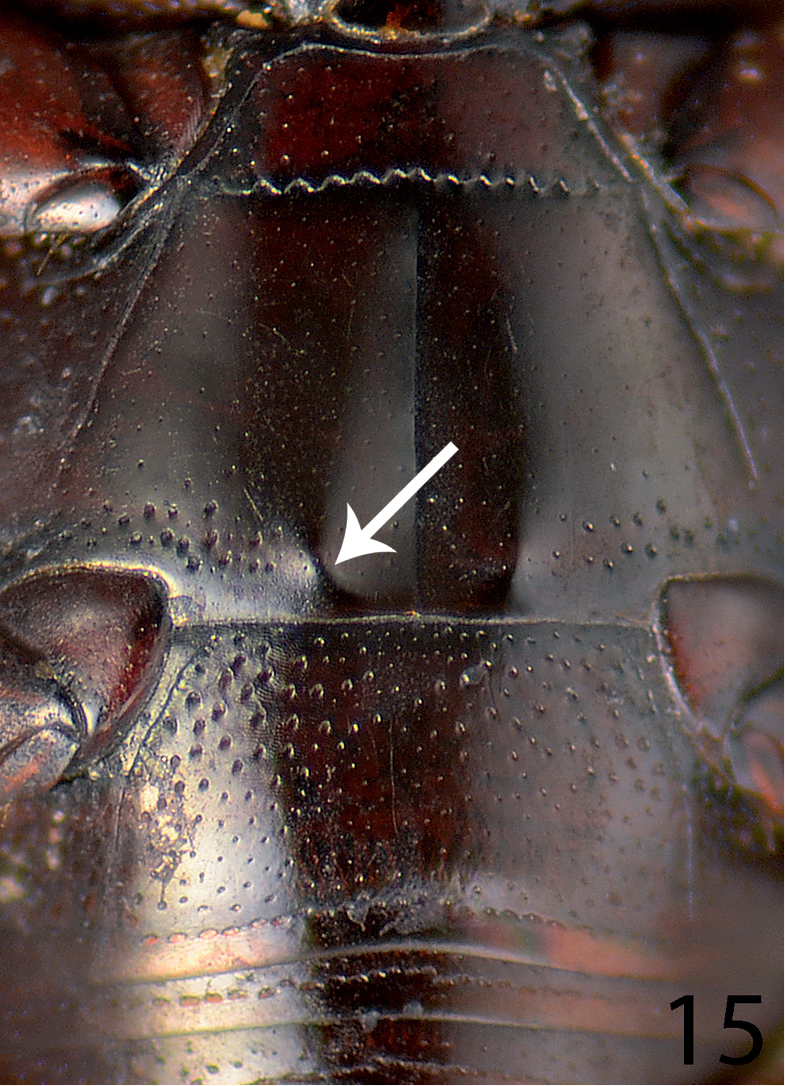
Saprinus (S.) calatravensis Fuente, 1899 metaventrite + abdomnen.

**Figure 16–24.**
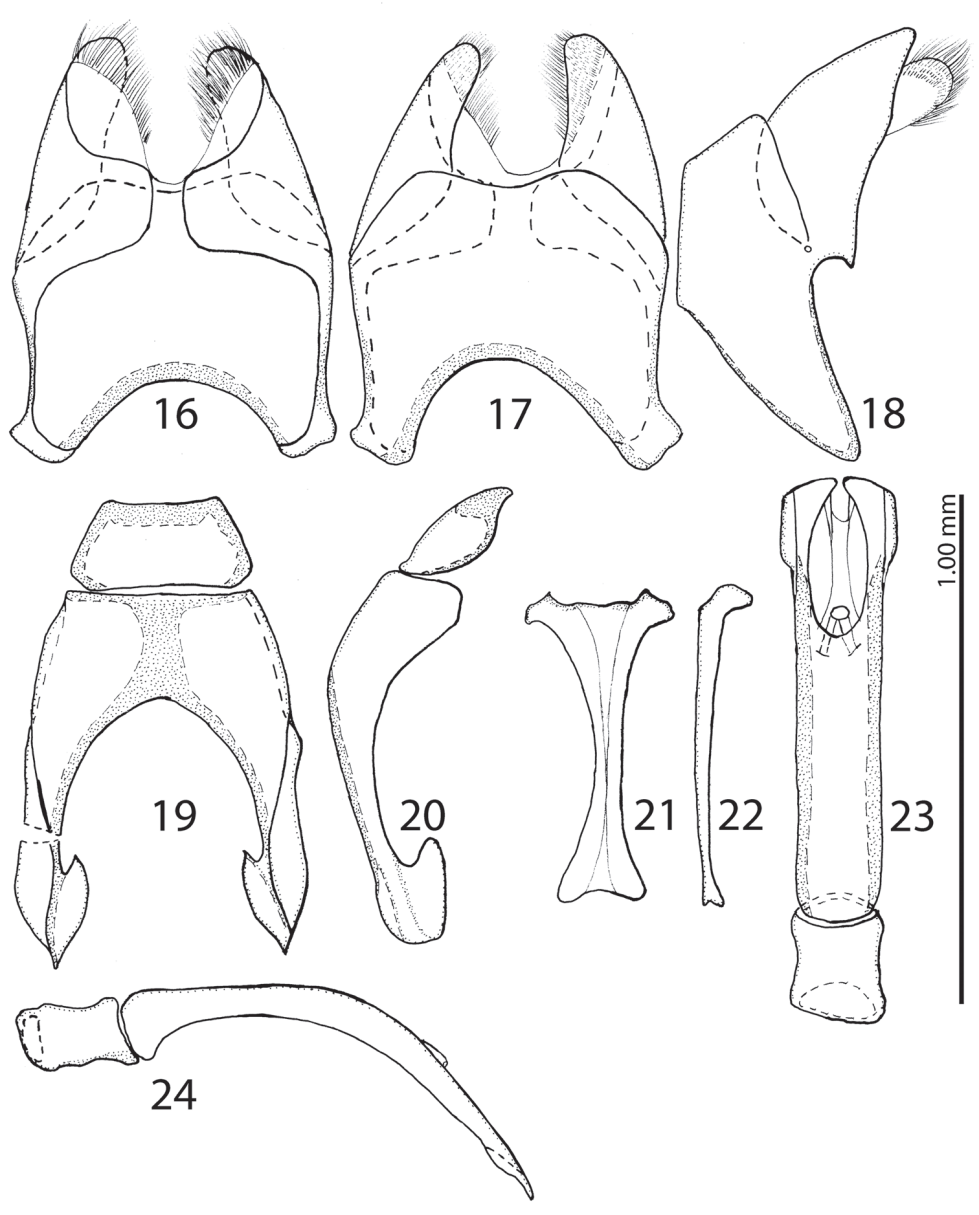
**16** Saprinus (S.) calatravensis Fuente, 1899 male genitalia: 8^th^ sternite and tergite, ventral view **17** 8^th^ sternite and tergite, dorsal view **18** 8^th^ sternite and tergite, lateral view **19** 9^th^ + 10^th^ tergites, dorsal view **20** 9^th^ + 10^th^ tergites, lateral view **21** spiculum gastrale (9^th^ sternite), ventral view **22** spiculum gastrale (9^th^ sternite), lateral view **23** aedeagus, dorsal view **24** aedeagus, lateral view.

**Figure 25.**
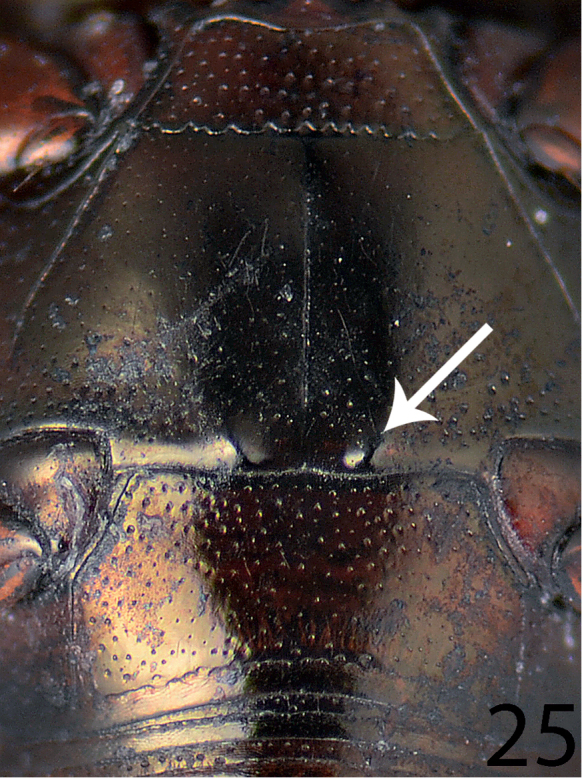
Saprinus (S.) chalcites (Illiger, 1807) metaventrite + abdomnen.

**Figures 26–34.**
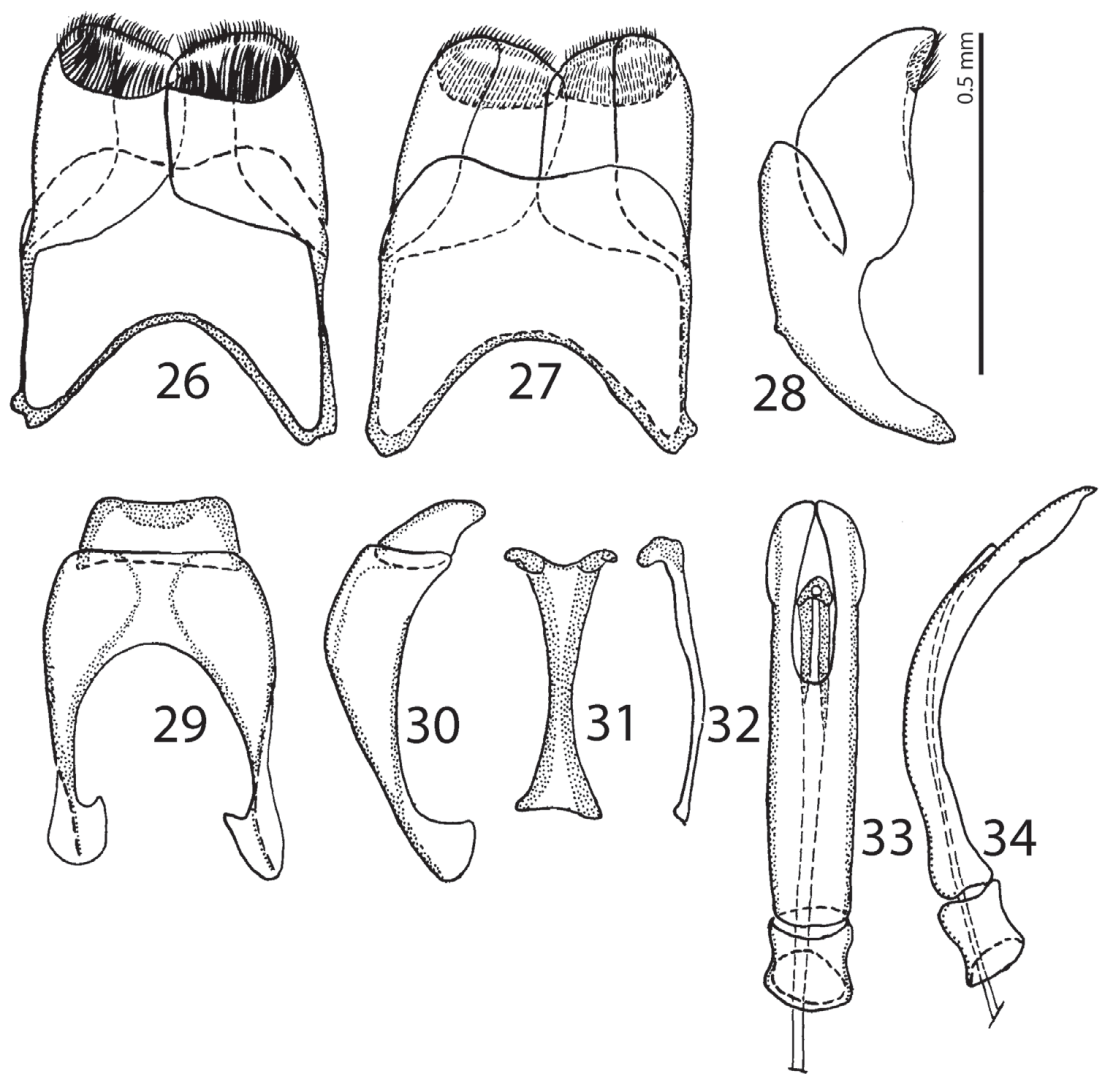
**26** Saprinus (S.) chalcites (Illiger, 1807) male genitalia: 8^th^ sternite and tergite, ventral view **27** 8^th^ sternite and tergite, dorsal view **28** 8^th^ sternite and tergite, lateral view **29** 9^th^ + 10^th^ tergites, dorsal view **30** 9^th^ + 10^th^ tergites, lateral view **31** spiculum gastrale (9^th^ sternite), ventral view **32** spiculum gastrale (9^th^ sternite), lateral view **33** aedeagus, dorsal view **34** aedeagus, lateral view.

**Figures 35–43.**
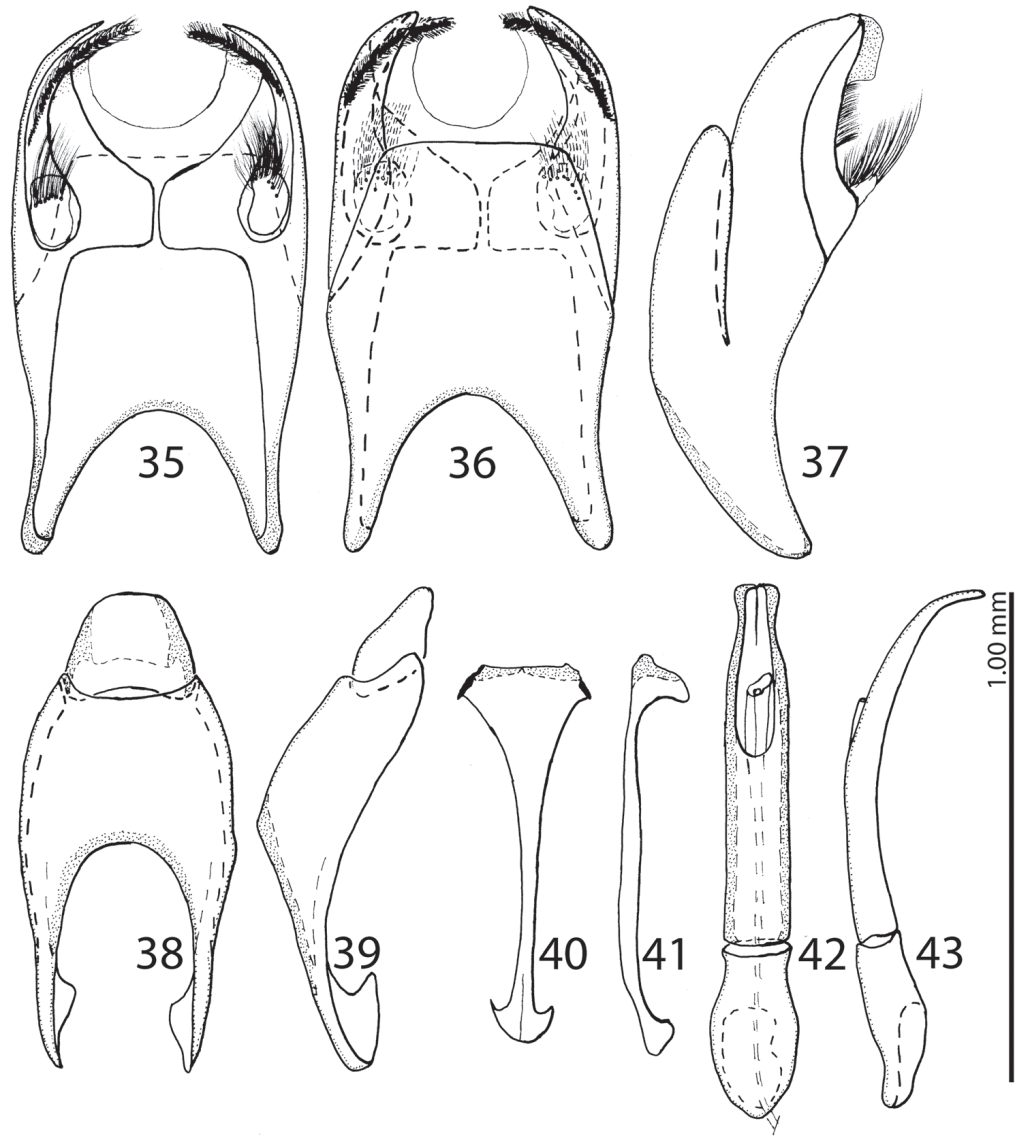
**35** Saprinus (S.) godet (Brullé, 1832) male genitalia: 8^th^ sternite and tergite, ventral view **36** 8^th^ sternite and tergite, dorsal view **37** 8^th^ sternite and tergite, lateral view **38** 9^th^ + 10^th^ tergites, dorsal view **39** 9^th^ + 10^th^ tergites, lateral view **40** spiculum gastrale (9^th^ sternite), ventral view **41** spiculum gastrale (9^th^ sternite), lateral view **42** aedeagus, dorsal view **43** aedeagus, lateral view.

**Figures 44–52.**
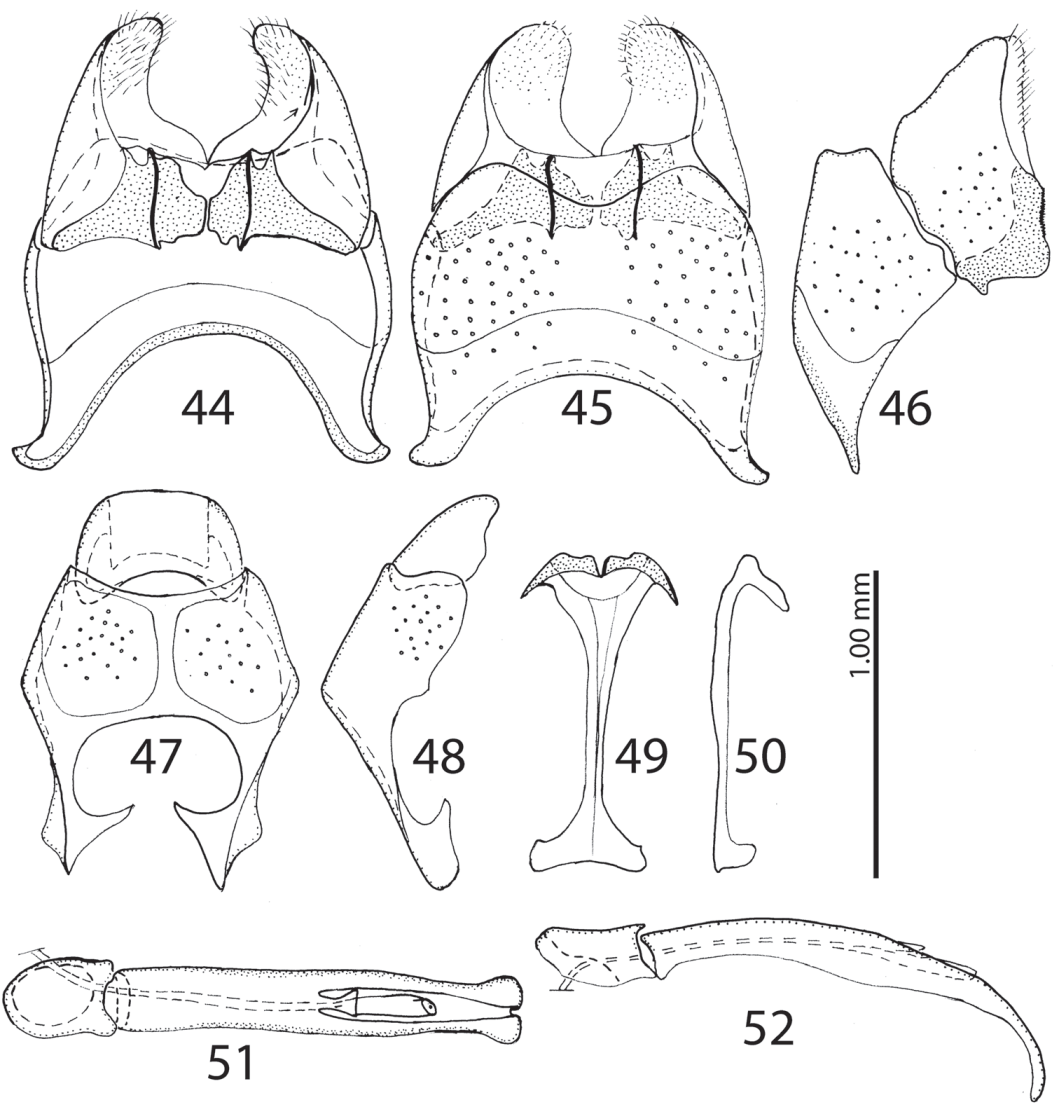
**44** Saprinus (S.) robustus Krása, 1944 male genitalia: 8^th^ sternite and tergite, ventral view **45** 8^th^ sternite and tergite, dorsal view **46** 8^th^ sternite and tergite, lateral view **47** 9^th^+10^th^ tergites, dorsal view **48** 9^th^+10^th^ tergites, lateral view **49** spiculum gastrale (9^th^ sternite), ventral view **50** spiculum gastrale (9^th^ sternite), lateral view **51** aedeagus, dorsal view **52** aedeagus, lateral view.

**Figure 53.**
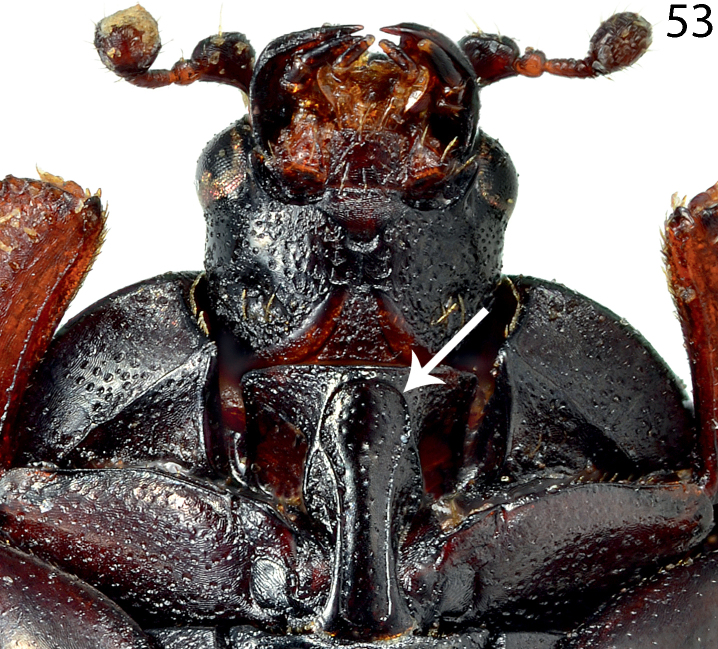
Saprinus (S.) submarginatus J. Sahlberg, 1913 prosternum.

**Figures 54–62.**
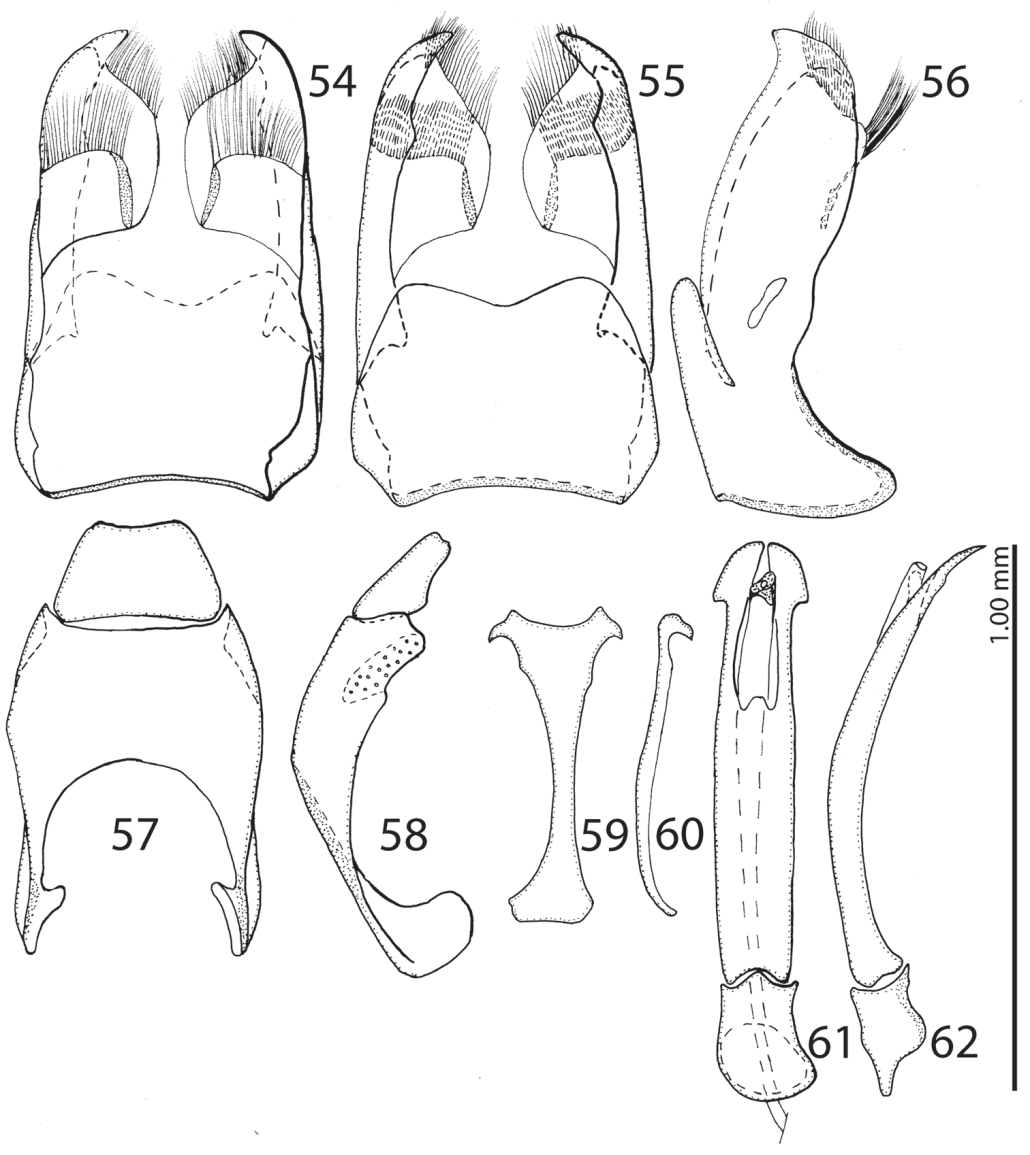
**54** Saprinus (S.) submarginatus J. Sahlberg, 1913 male genitalia: 8^th^ sternite and tergite, ventral view **55** 8^th^ sternite and tergite, dorsal view **56** 8^th^ sternite and tergite, lateral view **57** 9^th^+10^th^ tergites, dorsal view **58** 9^th^+10^th^ tergites, lateral view **59** spiculum gastrale (9^th^ sternite), ventral view **60** spiculum gastrale (9^th^ sternite), lateral view **61** aedeagus, dorsal view **62** aedeagus, lateral view.

**Figure 63.**
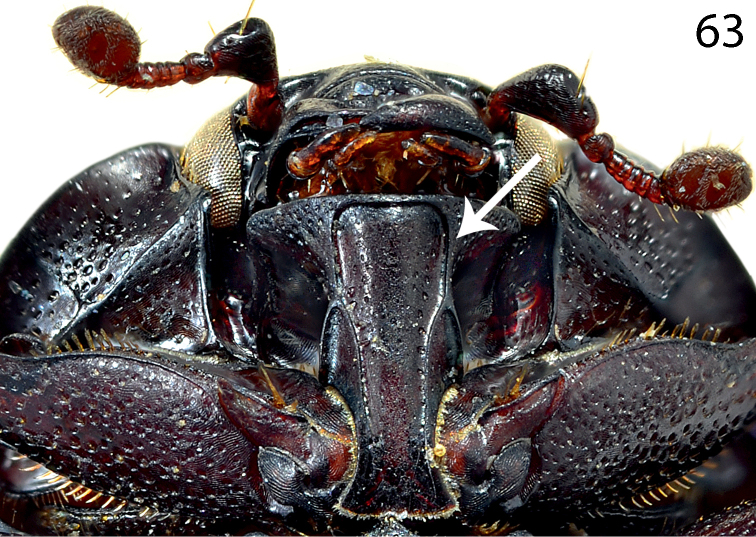
Saprinus (S.) subnitescens Bickhardt, 1909 prosternum.

**Figures 64–72.**
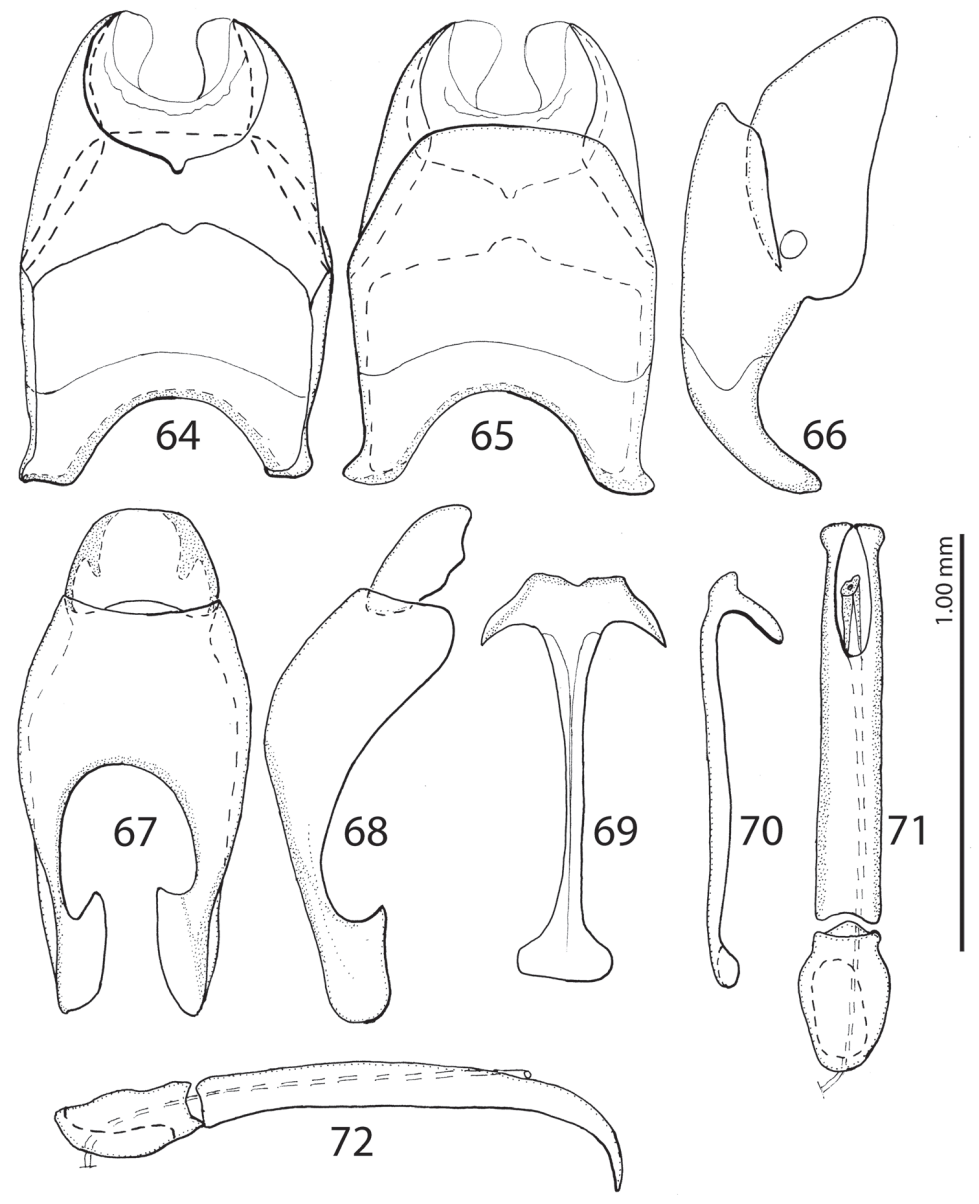
**64** Saprinus (S.) subnitescens Bickhardt, 1909 male genitalia: 8^th^ sternite and tergite, ventral view **65** 8^th^ sternite and tergite, dorsal view **66** 8^th^ sternite and tergite, lateral view **67** 9^th^ + 10^th^ tergites, dorsal view **68** 9^th^ + 10^th^ tergites, lateral view **69** spiculum gastrale (9^th^ sternite), ventral view **70** spiculum gastrale (9^th^ sternite), lateral view **71** aedeagus, dorsal view **72** aedeagus, lateral view.

**Figures 73–81.**
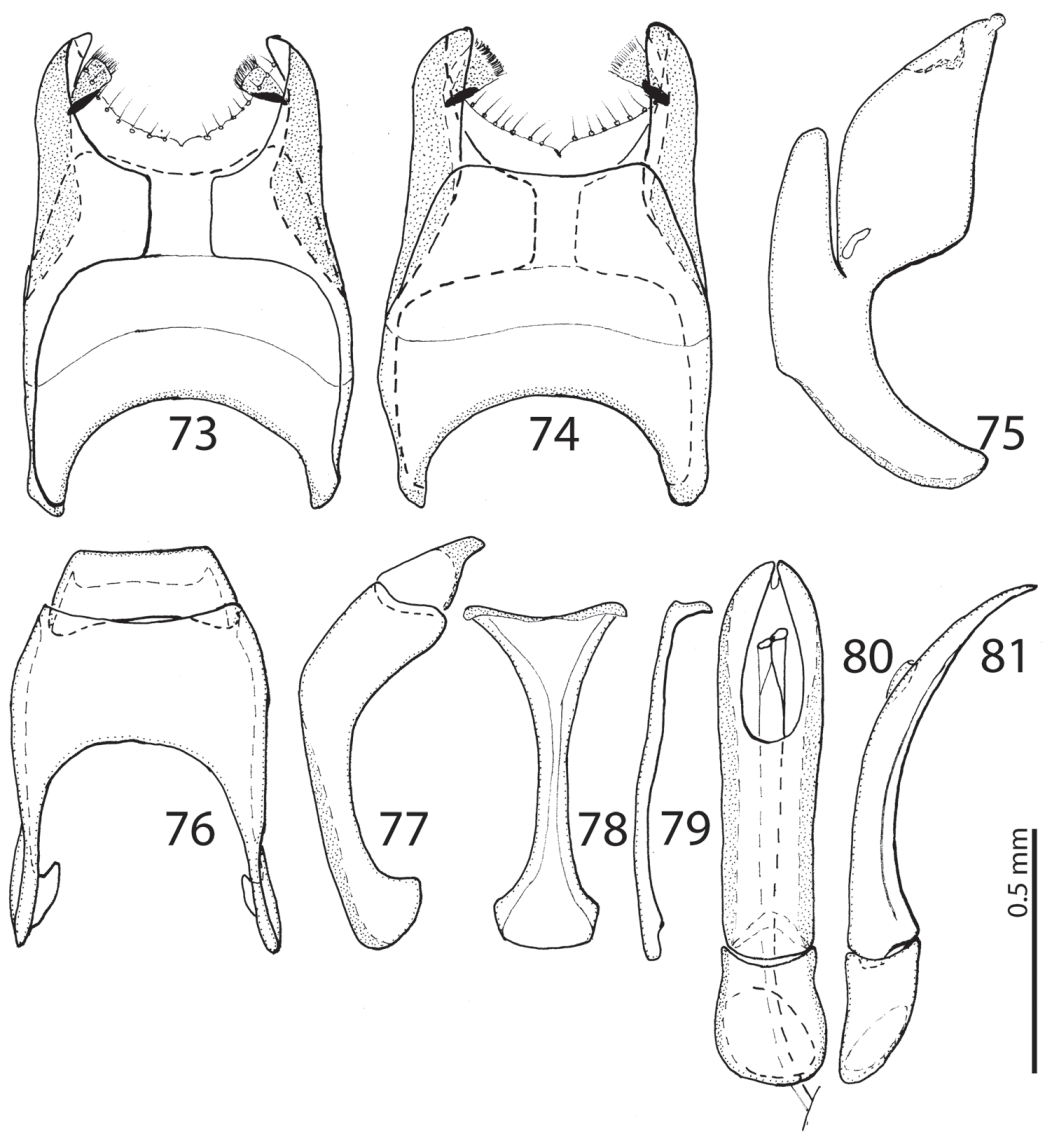
**73** Saprinus (S.) tenuistrius sparsutus Solsky, 1876 male genitalia: 8^th^ sternite and tergite, ventral view **74** 8^th^ sternite and tergite, dorsal view **75** 8^th^ sternite and tergite, lateral view **76** 9^th^ + 10^th^ tergites, dorsal view **77** 9^th^ + 10^th^ tergites, lateral view **78** spiculum gastrale (9^th^ sternite), ventral view **79** spiculum gastrale (9^th^ sternite), lateral view **80** aedeagus, dorsal view **81** aedeagus, lateral view.

**Figure 82.**
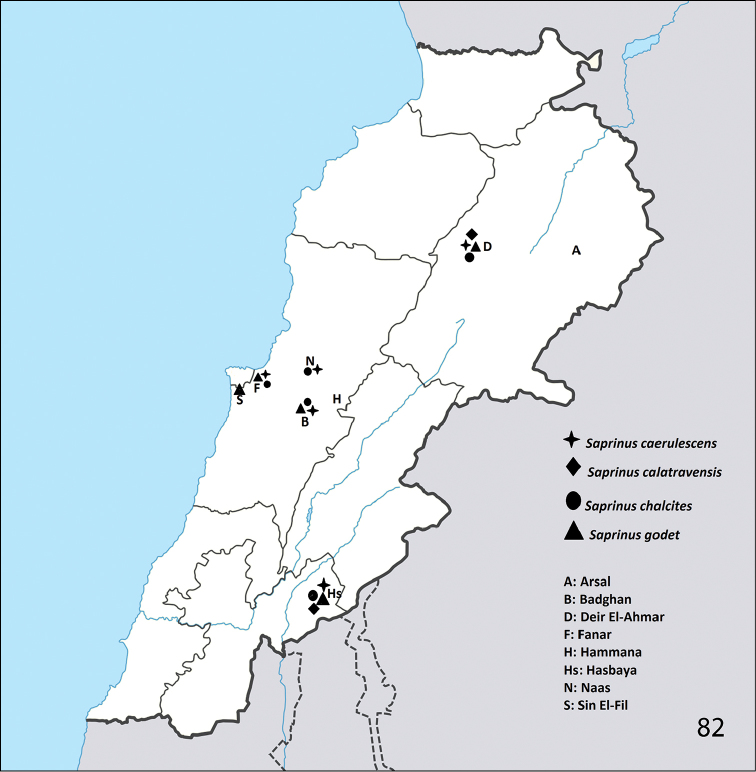
Distribution of S. (S.) caerulescens caerulescens, S. (S.) calatravensis, S. (S.) chalcites, and S. (S.) godet in Lebanon.

**Figure 83.**
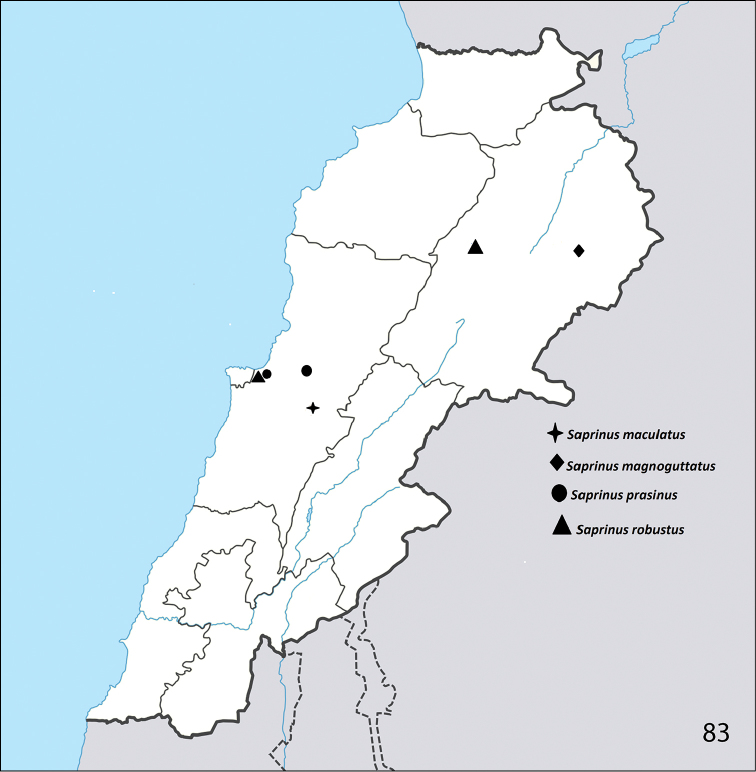
Distribution of S. (S.) maculatus, S. (S.) magnoguttatus, S. (S.) prasinus prasinus, and S. (S.) robustus in Lebanon.

**Figure 84.**
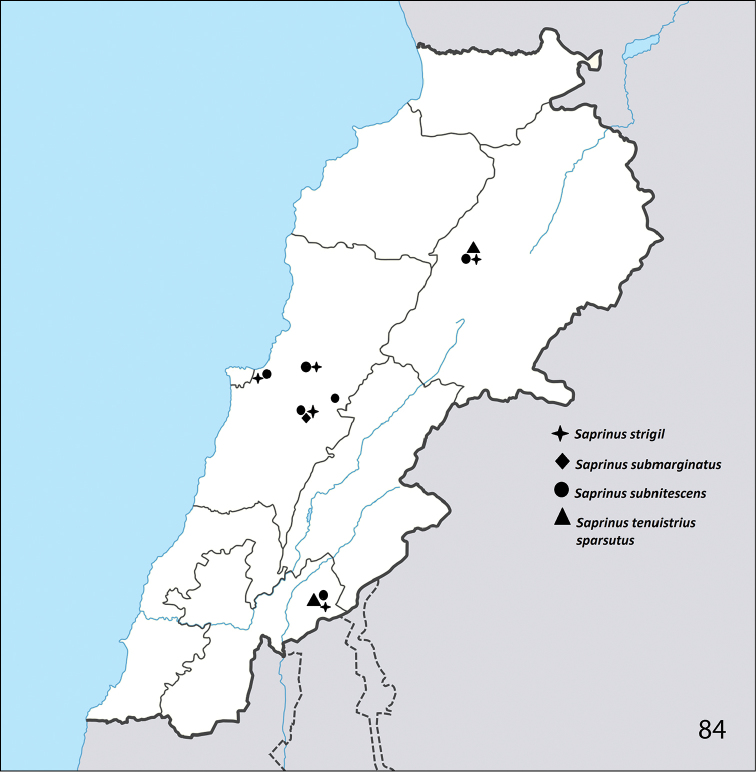
Distribution of S. (S.) strigil, S. (S.) submarginatus, S. (S.) subnitescens, and S. (S.) tenuistrius sparsutus in Lebanon.

**Figure 85.**
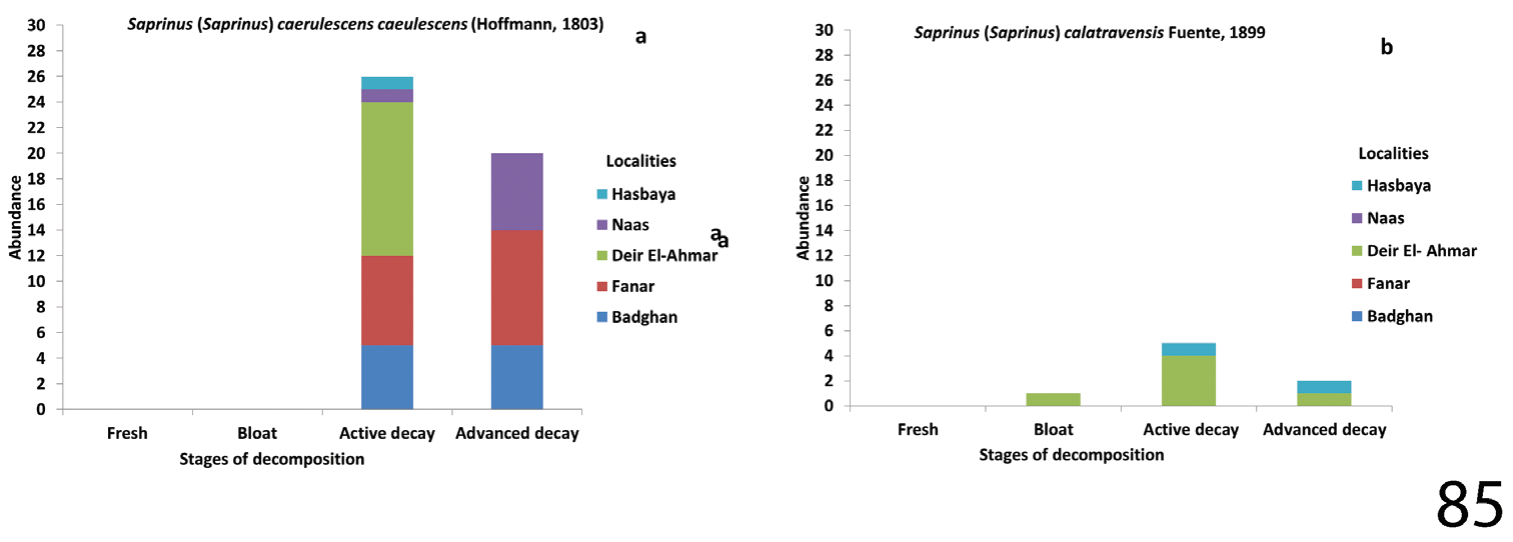
Abundance of Saprinus (S.) caerulescens caerulescens (85a), and S. (S.) calatravensis (85b) during the decomposition stages in different Lebanese localities.

**Figure 86.**
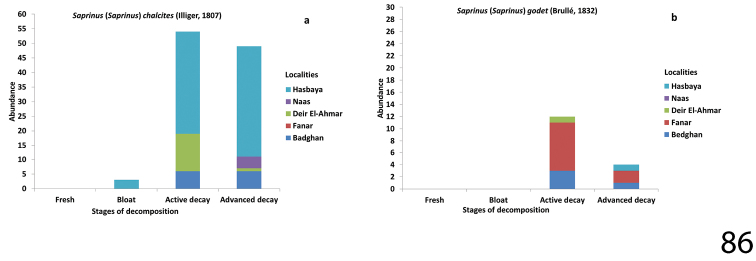
Abundance of Saprinus (S.) chalcites (86a) and S. (S.) godet (86b) during the decomposition stages in different Lebanese localities.

**Figure 87.**
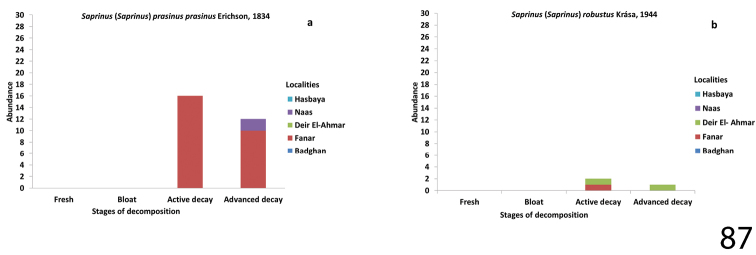
Abundance of Saprinus (S.) prasinus prasinus (87a) and S. (S.) robustus (87b) during the decomposition stages in different Lebanese localities.

##### Comment.

S. (S.) tenuistrius has another subspecies, the nominotypical S. (S.) tenuistrius tenuistrius Marseul, 1855, which is known from Egypt, north Africa, Ethiopia and Arabian Peninsula (Mazur 2011). According to Kryzhanovskij and Reichardt (1976) the nominotypical subspecies differs from the ssp. sparsutus by aciculate punctation on the apical elytral half (the subspecies sparsutus lacks this aciculate punctation).

### Association of the *Saprinus* species with the decomposing pig carcasses

**Figure 88.**
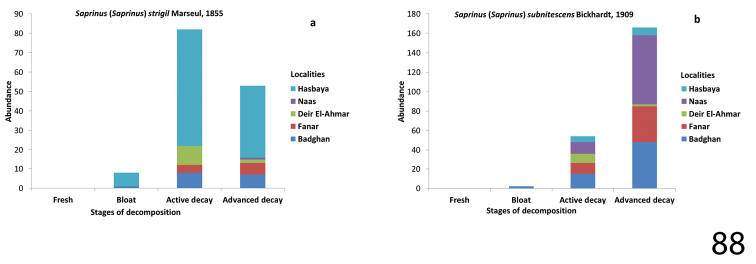
Abundance of Saprinus (S.) strigil (88a) and S. (S.) subnitescens (88b) during the decomposition stages in different Lebanese localities.

Saprinus (S.) subnitescens was collected during the sampling of the months March, April, May, June, July, August, and September. It was collected during three stages of decomposition; bloat, active decay and advanced decay. The maximum amount of specimens was observed during the advanced decay stage of the carcasses. This species was dominant (83 specimens) in Naas (Bikfaya) during spring season at the mean temperature of 16.4 °C. Specimens were collected during both active decay and advanced stages of decomposition from Naas, Badghan, and Fanar, and less frequently from Deir El-Ahmar and Hasbaya, respectively. Moreover, this species was also collected during the bloat stage of decomposition from Badghan (Fig. 88b).

Saprinus (S.) strigil was likewise collected during the sampling events of the months March, April, June, July, August, and September, with the maximum of 104 specimens collected in August from Hasbaya, when the mean temperature was 26.9 °C. The specimens were collected mainly during the active decay stage, less frequent during the advanced decay and minimally present during the bloat stages. Unlike Saprinus (S.) subnitescens, this species was rarely present in Naas. A few specimens were collected during the bloat stage from Badghan (Fig. 88a).

Saprinus (S.) chalcites was collected during the sampling events of the months of April, June, July, August, September and October. In general, it was dominant during the active decay stage of the carcasses. Peak abundance of 76 specimens was collected in August from Hasbaya during bloat, active decay and advanced decay stages of pig carcass decomposition. Similar amounts were collected during the active and advanced decay stages from Badghan. More specimens were collected during active decay stage than during the advanced decay stage from Deir El-Ahmar. S. (S.) chalcites was present in Naas only during the advanced decay stage (Fig. 86a).

Saprinus (S.) caerulescens caerulescens was captured in March, April, June, July and August; a maximum of 17 specimens was collected during the spring season from Fanar, when the mean weather temperature was 17.6 °C. In general, more specimens were collected during the active decay stages than those collected during the advanced decay ones. Similar amounts were collected during those stages from Badghan. It was collected during the decay stage mainly from Deir El-Ahmar and less frequently from Hasbaya. Regarding the locality Naas, this species was there found to be present mainly during the advanced stage of decomposition and less frequent during the active decay stage (Fig. 85a).

Saprinus (S.) prasinus prasinus – the peak of its abundance of 27 specimens was likewise in Fanar during spring (March and April). They were mainly captured during the active decay stage and found to be less numerous in the advanced decay stage. This species was likewise collected from Naas during same season during the advanced decay stage only (Fig. 87a).

Saprinus (S.) godet – the peak abundance of this species was in spring in Fanar. It is mainly present during the active decay stage of carcasses decomposition and in lesser numbers during the advanced decay stage. More specimens were collected during the active decay stage than from the advanced decay stage from Fanar and Badghan. It was only present during the active decay stage in Deir El-Ahmar and during the advanced decay stage in Hasbaya (Fig. 86b).

**Figure 89.**
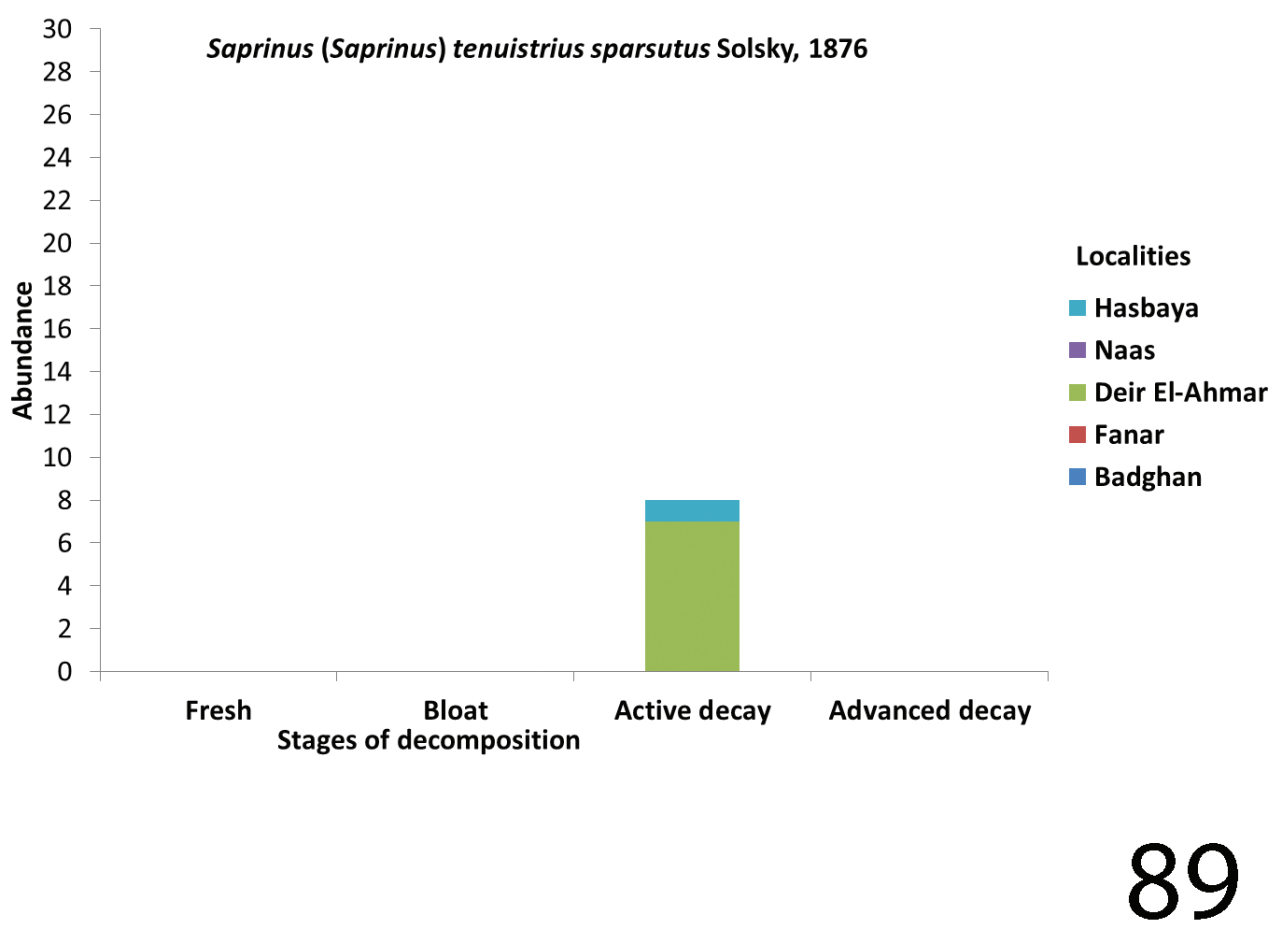
Abundance of Saprinus (S.) tenuistrius sparsutus during the decomposition stages in different Lebanese localities.

Both Saprinus (S.) tenuistrius sparsutus and Saprinus (S.) calatravensis were collected in July – August. The former was only collected during the active decay stages of Deir El-Ahmar and Hasbaya. It was more abundant in the locality Deir El-Ahmar in July; the mean temperature was 29.7 °C. The latter was mainly present during the active decay stage and less frequently during the bloat and the advanced decay stages (Figs 85b, 89).

Saprinus (S.) robustus was collected from Fanar in March during the active decay stage only, and from Deir El-Ahmar in July during both the active and advanced decay stages. Only a single specimen of Saprinus (S.) submarginatus and of Saprinus (S.) maculatus, respectively, was collected during the summer season from one locality (Badghan); the mean temperature was 20 °C. A male and a female specimen of Saprinus (S.) magnoguttatus were collected from a human corpse that was infested by Diptera larvae (Fig. 87b).

### Key to *Saprinus* Erichson, 1834 from Lebanon

This key includes only those species specifically recorded from Lebanon so far. For the *Saprinus* species found in the neighbouring countries (Israel, Palestine, Syria and Jordan), which are likely to occur also in Lebanon (see Lackner et al. 2015 for details), the reader is referred to the key of Kryzhanovskij and Reichardt (1976) that contains most or all of the possible Lebanese *Saprinus* species.

**Table:** 

1(4)	Elytra bicolored (Fig. 7)	**2**
2(3)	At least the entire lateral elytral margin orange-red, usually most part of the elytral disk orange-red with only a short band along the elytral suture black (Fig. 7)	**Saprinus (S.) maculatus (P. Rossi, 1790)**
3(2)	Each elytron with a well-defined red macula, never occupying the entire lateral elytral margin (Fig. 8)	**Saprinus (S.) magnoguttatus Reichardt, 1926**
4(1)	Elytra unicolored, never with red macula (Fig. 2)	**5**
5(6)	Pronotal hypomeron setose, fourth dorsal elytral stria strongly reduced, often absent. A large, usually metallic species (PEL = 5.00–7.50 mm) (Fig. 3)	**Saprinus (S.) caerulescens caerulescens (Hoffman, 1803)**
6(5)	Pronotal hypomeron asetose, fourth dorsal elytral stria usually not reduced, fully developed. Smaller species (PEL = 2.50–6.50 mm)	**7**
7(8)	Elytra, especially on their apical halves with very dense punctation, punctures aciculate and striolate, elytral intervals punctured, third dorsal elytral stria well-developed (Fig. 11)	**Saprinus (S.) strigil Marseul, 1855**
8(7)	Elytra with variously dense punctation, but punctures usually not aciculate or striolate (some specimens of S. (S.) robustus can have striolate punctures, but then the third dorsal elytral stria is always strongly reduced) (Fig. 2)	**9**
9(12)	Elytra with well-defined polished areas ‘mirrors’, punctation of elytral disk very dense, punctures separated by less than their own diameter, third dorsal elytral stria reduced to absent (Fig. 2)	**10**
10(11)	Elytral ‘mirror’ with microscopic scattered punctation, light to dark brown species, without greenish or bronze metallic hue, third dorsal elytral stria reduced, but usually discernible; elytral punctation in fourth elytral interval reaches elytral half (Fig. 2)	**Saprinus (S.) aegialius Reitter, 1884**
11(10)	Elytral ‘mirror’ glabrous, third dorsal elytral stria usually strongly reduced to absent, dorsum with distinct greenish or bronze metallic hue; punctation in fourth elytral interval does not reach elytral half (Fig. 9)	**Saprinus (S.) prasinus prasinus Erichson, 1834**
12(9)	Elytra without well-defined polished areas (‘mirrors’), punctation of the elytral disk less dense, punctures usually separated by their own diameter or more (Fig. 13)	**13**
13(16)	Apices of carinal prosternal striae strongly divergent, laying on lateral sides of the prosternal process (Fig. 63); usually larger species (PEL = 3.50–5.30 mm)	**14**
14(15)	Pronotal depressions deep, third dorsal elytral stria usually not reduced, light to dark brown species with slight bronze metallic hue (Fig. 13), male with deeply depressed metaventrite; male terminalia: apex of 8^th^ sternite (velum) asetose, 8^th^ sternite medially not strongly sclerotized (Figs 64–72)	**Saprinus (S.) subnitescens Bickhardt, 1909**
15(14)	Pronotal depressions shallow, third dorsal elytral stria usually strongly reduced, black species without metallic hue (Fig. 10), male with only shallowly depressed metaventrite; male terminalia: apex of 8^th^ sternite (velum) with dense tiny setae, 8^th^ sternite medially strongly sclerotized (Figs 44–52)	**Saprinus (S.) robustus Krása, 1944**
16(13)	Apices of prosternal striae divergent, but never laying on lateral sides of the pronotal process (Fig. 53); usually smaller species (PEL = 2.50–3.90 mm)	**17**
17(18)	Pronotal depressions absent, pronotal disk medially with distinct punctation, humeral elytral stria confluent with inner subhumeral one creating a supplementary dorsal elytral stria parallel to first (Fig. 6); male terminalia: apices of 8^th^ sternite with thin, dense brush of setae, medio-laterally with a bean-shaped setose sclerite, aedeagus strongly constricted before apex (Figs 35–43)	**Saprinus (S.) godet (Brullé, 1832)**
18(17)	Pronotal depressions present, pronotal disk medially with only scattered fine punctation (Fig. 14)	**19**
19(20)	Entire elytral disk with punctation, punctures separated by twice or more their diameter, dorsal elytral striae thin, impunctate (Fig. 14), antennal club large, light-amber coloured; male terminalia: apices of 8^th^ sternite with tiny triangular accessory sclerite furnished with micro-setae, aedeagus short and stout, not dilated apically (Figs 73–81)	**Saprinus (S.) tenuistrius sparsutus Solsky, 1876**
20(19)	At least the area between united sutural and fourth elytral striae without punctation (or punctures microscopic), punctures of elytral disk separated usually by less than twice their diameter (Fig. 12), antennal club medium-sized, reddish-brown. The following species are usually only reliably identifiable based on their male terminalia	**21**
21(22)	Apical margin of metaventrite of male without tubercles. Male terminalia: 8^th^ sternite with two rows of brush-like setae: one situated approximately medially and another apically, aedeagus constricted before apex; apex rounded (Figs 54–62) (Fig. 12)	**Saprinus (S.) submarginatus J. Sahlberg, 1913**
22(21)	Apical margin of metaventrite of male with two distinct tubercles (Fig. 15)	**23**
23(24)	Tubercles on the apical margin of metaventrite of male slightly removed from metaventral margin (Fig. 15). Dorsal elytral striae surpassing elytral half; male terminalia: 8^th^ sternite with large setose velum (best seen especially from lateral view), apex of aedeagus rectangularly dilated, truncated (Figs 16–24) (Fig. 4)	**Saprinus (S.) calatravensis Fuente, 1899**
24(23)	Tubercles situated almost on the very apical metaventral margin (Fig. 25); dorsal elytral striae usually not surpassing elytral half; male terminalia: 8^th^ sternite without large setose velum, apex of aedeagus only slightly roundly dilated (Figs 26–34) (Fig. 5)	**Saprinus (S.) chalcites (Illiger, 1807)**

## Discussion

In an experiment performed in Al-Baha Province (Kingdom of Saudi Arabia) *Saprinus* species were commonly found on rabbit carcasses during spring, summer, and autumn. In spring, they were found to be abundant during the active decay and the first two days of the dry decay stages. However, in autumn they were common ranging from the bloat stage to the dry decay stages (Abouzied 2014). Based on Sawaby et al. (2016), in a study that was carried out in Egypt, the authors stated the usefulness of the Histeridae in forensic investigations as they help in time of death estimation. According to Kovarik and Caterino (2016) the majority of histerids are thermophilic, and warmer months of the year correspond to their peak abundance. In general, carcasses decomposition is faster during the summer, due to the large number of insects attracted, whereas rainfall may lead to delayed oviposition and pupation (Abd El-Bar et al. 2016). Based on Bala and Kaur (2014), in an experiment performed on buried pig carcasses in India, *Saprinus* species and other histerids were common throughout the experiment. Saprinus (S.) semistriatus was previously mentioned in forensic-based experiments as being collected regularly on carrion especially during the active decay stage and useful in post-mortem interval (PMI) estimation (Szelecz et al. 2018). Saprinus (S.) chalcites was recorded on rabbit carcasses during insect succession study in Al-Ahsaa Oasis (Kingdom of Saudi Arabia) in summer, winter, and spring (Shaalan et al. 2017). Saprinus (S.) aeneus was recorded on human cadaver in Italy and utilised to establish the PMI (Introna et al. 1998). Our data concerning carrion-baited *Saprinus* reflect different peak abundances among species. Generally speaking, the majority of studied species show their peak abundance during the warmer months of the year.

In our survey, Saprinus (S.) caerulescens, Saprinus (S.) godet, Saprinus (S.) prasinus, and Saprinus (S.) robustus were attracted to cadavers during the both active decay and advanced decay stages of decomposition, when the Diptera larvae are active and feeding on the carcasses, and were also recovered from the soil after the departure of Diptera larvae to pupate. Saprinus (S.) strigil, Saprinus (S.) subnitescens, Saprinus (S.) *calatravensis*, and Saprinus (S.) chalcites were present on the carcasses earlier, in the bloat stage in the summer season, whereas in the spring months (as observed in Fanar and Naas) the arrival of *Saprinus* on the carcass was during the active decay stage (Figs 85, 86, 87, 88). Our data suggest that the active decay stage was reached faster during the summer season than during the spring. The histerid beetles were frequent on all carcasses, but were found to be more diverse during the warm season rather than during autumn. According to our observations, histerids tend to stay in the soil underneath the carcass during the day and become active during the night. Some are predacious not only on Diptera immature stages but also on the dermestid larvae (Byrd and Castner 2000). Moreover, necrophagous beetles such as Dermestidae are present during the end of the decay process while predators are attracted earlier during the decomposition process (Santos and Santos 2016). This could explain the presence of *Saprinus* during the advanced decay when the dermestids are active. However, the decrease in the abundance of *Saprinus* during the late stages of decomposition coincides with the increase of *Dermestes* immature stages. The other histerid specimens among the genera *Margarinotus*, *Atholus* and *Hypocacculus* were also reported during the decomposition process of the carcasses, but with lower abundance in comparison with *Saprinus*. *Margarinotus*, *Atholus* as well as *Hypocacculus* are known to be attracted to carrion (Kovarik and Caterino 2016).

It is possible that different patterns of residency on carrion in *Saprinus* result from the differences in release patterns of the volatile organic compounds (VOCs) attracting them (Bajerlein et al. 2011). The decrease in the abundance in late decay stages could be due to the decrease in VOCs. In forensic entomology, Coleoptera is an order of great importance; however, the neglect of this order is due to difficulties regarding taxonomy or its role on the carrion (Almeida et al. 2015). Each biogeographic region has its specific carrion insect fauna (Santos and Santos 2016). Our survey shows the diversity of *Saprinus* in Lebanon, their feeding habits and peak abundances during the warm season in the country.

In the present study, 489 specimens of *Saprinus* were recorded, representing 13 species. Out of these, 8 are new records for the Lebanese fauna (Fig. 1). The main aim of this study was to monitor the activity of these beetles on cadavers and identify the species that can be used in later forensic investigations of the country. To ease the identifications, a key for the species is provided, accompanied with illustrations of habitus and male genitalia. More rigorous research concerning both taxonomic identification and the larval development should be conducted to clearly establish the importance of histerid beetles in criminal investigations.

## Supplementary Material

XML Treatment for Saprinus (Saprinus) aegialius

XML Treatment for Saprinus (Saprinus) caerulescens caerulescens

XML Treatment for Saprinus (Saprinus) calatravensis

XML Treatment for Saprinus (Saprinus) chalcites

XML Treatment for Saprinus (Saprinus) godet

XML Treatment for Saprinus (Saprinus) maculatus

XML Treatment for Saprinus (Saprinus) magnoguttatus

XML Treatment for Saprinus (Saprinus) prasinus prasinus

XML Treatment for Saprinus (Saprinus) robustus

XML Treatment for Saprinus (Saprinus) strigil

XML Treatment for Saprinus (Saprinus) submarginatus

XML Treatment for Saprinus (Saprinus) subnitescens

XML Treatment for Saprinus (Saprinus) tenuistriussparsutus
